# Molecular Profiles, Treatment-Associated Survival, and Prognosis in Early-Onset Versus Late-Onset Gastric Cancer: A Systematic Review and Meta-Analysis

**DOI:** 10.3390/jcm15207814

**Published:** 2026-10-10

**Authors:** Jianbin Wen, Linyong Zhao

**Affiliations:** 1 Department of General Surgery, West China Hospital, Sichuan University, Chengdu 610041, China; 2 Laboratory of Gastric Cancer, State Key Laboratory of Biotherapy, Collaborative Innovation Center of Biotherapy and Cancer Center, West China Hospital, Sichuan University, Chengdu 610041, China; 3 Gastric Cancer Center, West China Hospital, Sichuan University, Chengdu 610041, China

**Keywords:** early-onset gastric cancer, molecular profiles, treatment-associated survival, prognosis, meta-analysis

## Abstract

**Background:** Whether early-onset gastric cancer (EOGC) represents a distinct clinical entity requiring age-specific management remains uncertain. We synthesized molecular profiles, treatment-associated survival, and prognosis to determine what current evidence supports in practice and requires prospective validation. **Methods:** This PROSPERO-registered, PRISMA-compliant review searched four databases (PubMed, Web of Science Core Collection, Embase, and the Cochrane Library) from 1990 to 2025. Random-effects models compared EOGC with late-onset gastric cancer (LOGC) using each study’s reported age definition, provided that the upper age cutoff for EOGC did not exceed 50 years, and evaluated treatment–survival associations within EOGC. Robustness was evaluated by restricted maximum likelihood (REML) estimation with Knapp–Hartung adjustment, subgroup analyses, meta-regression, and prediction intervals; certainty was assessed using Grading of Recommendations Assessment, Development and Evaluation (GRADE). **Results:** Fifty-eight studies involving 194,671 patients were included. EOGC showed lower observed odds of human epidermal growth factor receptor 2 (HER2) positivity (odds ratio [OR], 0.66; 95% confidence interval [CI], 0.53–0.84) and microsatellite instability (MSI)/deficient mismatch repair (dMMR) positivity (OR, 0.33; 95% CI, 0.12–0.91); p53 protein overexpression was less frequent, whereas the lower tumor protein p53 (TP53) mutation frequency represented only a non-significant numerical trend. E-cadherin loss was more frequent only under uniform immunohistochemical criteria (OR, 1.82; 95% CI, 1.16–2.85). These largely unadjusted molecular estimates indicate prevalence patterns rather than independent age effects. Radical resection was associated with lower mortality (hazard ratio [HR], 0.37; 95% CI, 0.22–0.61), but confounding by indication and a prediction interval crossing unity precluded causal inference. The chemotherapy association was not retained under REML–Knapp–Hartung analysis. Evidence was insufficient to establish differences in overall, disease-free, or cancer-specific survival. Certainty was low to very low. **Conclusions:** Age alone does not justify altered biomarker testing, systemic treatment, or surveillance. This review distinguishes actionable practice from unsupported extrapolation: established biomarker testing should be applied irrespective of age, and young age should not deter assessment for curative-intent resection. It also defines the evidence standards required before any EOGC-specific stratification can be adopted.

## 1. Introduction

Gastric cancer remains a major global health burden, with an estimated 968,000 new cases and 660,000 deaths in 2022 [1]. Although gastric cancer predominantly affects older adults, recent evidence indicates an increasing incidence among younger individuals. Early-onset gastric cancer (EOGC) accounts for approximately 4.6–14.8% of newly diagnosed gastric cancers [2], although proportions exceeding 30% have been reported in some regions [3]. This epidemiological shift has prompted investigation into whether EOGC represents a clinically and biologically distinct form of gastric cancer.

EOGC is defined by age at diagnosis, but no universally accepted cutoff exists, with thresholds of 50 [4], 45 [5], and 40 years [6] applied across studies. Compared with late-onset gastric cancer (LOGC), EOGC is more frequently diagnosed in women and in the non-cardia stomach, more often presents symptomatically, and is associated with diffuse-type, poorly differentiated or undifferentiated, and signet-ring cell histology. A family history of gastric cancer has also been associated with EOGC [7]. Collectively, these clinicopathological features raise the possibility that the pathogenesis of EOGC differs from that of LOGC.

Molecular studies have also identified potential differences between EOGC and LOGC, although the findings remain inconsistent. Human epidermal growth factor receptor 2 (HER2) warrants particular attention because it is an established predictive biomarker for HER2-directed therapy in advanced gastric cancer. Some studies have reported lower HER2 positivity in EOGC [4,8], whereas others have found no significant difference [9,10]. Similarly, microsatellite instability (MSI)/deficient mismatch repair (dMMR) has been reported less frequently in EOGC [9,11], although this association has not been consistently observed [4,8]. Conflicting results have also been reported for p53 protein overexpression and tumor protein p53 (TP53) mutation, as well as for CDH1 alterations and E-cadherin loss [9,12,13,14]. These inconsistencies may reflect variation in age cutoffs, biomarker assays, histological composition, and population characteristics.

The therapeutic implications of EOGC also remain uncertain. Because EOGC is often diagnosed at a more advanced TNM stage and is associated with diffuse-type histology, the use of more intensive treatment strategies has been considered. However, the survival associations of radical resection and chemotherapy, specifically in patients with EOGC, remain poorly characterized. Studies evaluating radical resection in EOGC have yielded inconsistent results, with some reporting better survival and others finding no clear association. Although younger patients may better tolerate intensive treatment because they generally have fewer comorbidities and greater physiological reserve, any potential survival advantage may be attenuated by adverse tumor biology. Accordingly, whether EOGC is independently associated with overall survival (OS), disease-free survival (DFS), or cancer-specific survival (CSS) remains uncertain [15,16,17,18].

Despite growing interest in EOGC, no comprehensive synthesis has integrated its molecular profile, treatment–survival associations, and prognostic outcomes. The available evidence is derived largely from retrospective, geographically concentrated studies that use heterogeneous age cutoffs and biomarker assessment methods, precluding firm conclusions regarding whether EOGC warrants distinct clinical management. Although previous meta-analyses have separately examined the molecular and clinicopathological features of EOGC, none have simultaneously synthesized treatment–survival associations and prognostic outcomes across multiple survival endpoints. We therefore conducted a systematic review and meta-analysis to (1) compare key molecular biomarkers, including HER2, MSI/dMMR, EBV, p53 protein overexpression, and TP53 mutation and E-cadherin, between EOGC and LOGC; (2) evaluate the associations of radical resection and chemotherapy with survival in EOGC; and (3) compare OS, DFS, and CSS between EOGC and LOGC. By integrating molecular, treatment, and survival evidence within a single framework, this meta-analysis provides a more comprehensive assessment of the clinicobiological characteristics and clinical relevance of EOGC. It also evaluates whether the available evidence supports the age-specific management of EOGC and identifies priorities for prospective validation.

## 2. Methods

The review protocol was registered in PROSPERO (CRD420251207966) on 10 November 2025, after a pilot search conducted on 1 November 2025 but before formal study screening, data extraction, or statistical analysis. The full protocol is publicly available through the PROSPERO registry, and the review was conducted and reported in accordance with PRISMA [19]. The completed PRISMA 2020 checklist is provided in Supplementary Materials (Table S11).

The protocol prespecified the primary molecular markers (HER2, PD-L1, CLDN18.2, and E-cadherin), treatment analyses (neoadjuvant therapy, surgical type, and adjuvant therapy), and prognostic outcomes (overall survival, disease-free survival, and 5-year survival); subgroup analyses by age, sex, and cancer stage were planned, as were sensitivity analyses based on heterogeneity. Protocol deviations comprised the addition of MSI/dMMR, Epstein–Barr virus (EBV), p53 protein overexpression, and TP53 mutation analyses when relevant data became available; omission of programmed death-ligand 1 (PD-L1) and claudin 18.2 (CLDN18.2) because extractable data were insufficient; post hoc subgroup analyses by region, detection method, and study population; exploratory univariate and multivariable meta-regression of overall survival; and omission of the prespecified sex- and stage-based subgroup analyses because stratum-specific data were inconsistently reported. All other methods followed the registered protocol. Because several modifications were data-driven, all deviations are reported transparently to permit assessment of their potential influence on the findings.

### 2.1. Search Strategy

PubMed, Web of Science Core Collection, Embase, and the Cochrane Library were systematically searched for studies published from 1 January 1990, to 1 November 2025. A meta-analysis published online after search closure was discussed as contextual evidence but was not eligible for inclusion in the quantitative synthesis [20]. No language or geographic restrictions were applied; non-English records were translated using DeepL (Cologne, Germany) for screening and full-text assessment. EOGC was primarily defined as gastric cancer diagnosed at age ≤50 years; studies that used lower but clearly defined age cutoffs, such as 30, 40, or 45 years, were also eligible. Controlled vocabulary and free-text terms for gastric cancer, including “stomach neoplasms” and “gastric cancer,” were combined with age-related terms, including “young” and “early onset.” Non-original publications, including reviews, meta-analyses, and case reports, were excluded. The complete search strategies for all four databases—including platforms, controlled vocabulary, field codes, date limits, and the search date—are provided in Table S1 to support reproducibility. Prior to final publication, the search will be re-run to identify any additional eligible studies published after the original search date.

Three complementary domains were evaluated: molecular characteristics, treatment-associated survival, and age-related prognosis.

For the molecular domain, biomarker prevalence was compared between EOGC and LOGC according to the age definitions reported in the individual studies. Eligible studies reported odds ratios (ORs) with 95% confidence intervals (CIs) or raw data sufficient for calculation.

For the treatment domain, the objective was to estimate the associations of radical resection and chemotherapy with overall survival among patients with EOGC. Radical resection was defined as microscopically margin-negative (R0) gastrectomy with curative intent, with non-radical, palliative, or no resection serving as the comparator. Chemotherapy was analyzed as a single exposure category because the included studies did not distinguish treatment settings (neoadjuvant, adjuvant) and further stratification was not feasible. Eligible studies provided adjusted hazard ratios (HRs) from multivariable Cox models or HRs reconstructed from survival curves derived from propensity-score matching, other matched designs, inverse probability weighting, or multivariable adjustment.

For the prognostic domain, the objective was to compare overall survival, disease-free survival, and cancer-specific survival between EOGC and LOGC. Eligible studies provided adjusted or design-adjusted HRs with 95% CIs.

### 2.2. Inclusion and Exclusion Criteria

Qualified studies fulfilled these conditions: (1) patients had pathologically confirmed gastric cancer; (2) EOGC was defined using a clearly specified upper age cutoff that did not exceed 50 years (e.g., ≤30, ≤40, ≤45, or ≤50 years); (3) the study reported ORs with 95% CIs, raw data sufficient to calculate ORs for molecular markers, adjusted HRs with 95% CIs for treatment or survival outcomes, or survival curves derived from adjusted, propensity-score-matched, matched, or weighted analyses that allowed HR reconstruction; and (4) the study design was cohort or case–control.

No language restrictions were applied during eligibility assessment. Non-English records were translated using DeepL (Cologne, Germany) to facilitate screening and full-text evaluation, thereby ensuring that language was not a basis for exclusion.

Studies were excluded if they: (1) used an EOGC cutoff >50 years or did not clearly define the cutoff; (2) were reviews, meta-analyses, case reports, commentaries, letters, guidelines, conference abstracts, animal studies, or basic research; (3) lacked relevant molecular, treatment, or survival data; (4) focused exclusively on specific gastric cancer subtypes, such as neuroendocrine carcinoma, hereditary diffuse gastric cancer, or signet-ring cell carcinoma; (5) reported duplicate populations; (6) included fewer than 10 patients; (7) had objectives unrelated to this review; or (8) lacked full text or extractable effect estimates, or reported only unadjusted HRs, univariable Cox estimates, log-rank *p* values, or unmatched/unadjusted Kaplan–Meier curves for treatment or prognostic outcomes.

### 2.3. Data Extraction and Quality Assessment

All retrieved records were imported into EndNote for management. Two reviewers independently screened titles, abstracts, and full texts against the eligibility criteria. Disagreements were resolved through discussion and, when necessary, repeat independent assessment. The same two reviewers then independently extracted data. The data extraction form was piloted on five randomly selected studies and refined prior to full implementation. When necessary, corresponding authors were contacted to request missing or clarifiable information. The following study-level characteristics were independently extracted: first author, publication year, country, data source, sample size, EOGC age cutoff, molecular marker status, assay methods, diagnostic criteria, treatment variables, and adjusted or extractable HRs with 95% CIs for survival outcomes.

For treatment and prognostic outcomes, the primary extraction hierarchy was as follows: multivariable-adjusted HRs were extracted preferentially; if these were unavailable, HRs were reconstructed only from survival curves generated after propensity-score matching, other matched designs, inverse probability weighting, or multivariable adjustment. Only one included study (Deng et al., 2025) [21] required HR reconstruction; its survival curve was derived from a propensity-score-matched cohort and constituted a conventional Kaplan–Meier curve; all other effect estimates were directly extracted from published multivariable Cox regression models or from Cox analyses performed after propensity-score matching. Curves based on unmatched or unadjusted cohorts were not used for HR reconstruction in the main analyses. For eligible adjusted or design-adjusted survival curves, time-to-event information was extracted using Engauge Digitizer version 12.1, and log HRs with standard errors were estimated according to Tierney et al. [22]; detailed procedures for HR reconstruction are provided in the Supplementary Methods. If multiple HRs from the same study compared different EOGC age subgroups with a common LOGC reference group, they were first combined within the study using an inverse-variance fixed-effect model to avoid double-counting before inclusion in the overall meta-analysis. This approach assumed independence between estimates sharing a common reference group but avoided arbitrary selection of a single age subgroup. Its influence was examined in sensitivity analyses excluding correlated estimates (Table S9).

Study quality was assessed independently by two reviewers using the Newcastle–Ottawa Scale (NOS), which evaluates selection, comparability, and outcome assessment, with scores ≥ 7 indicating high quality. While NOS provides a pragmatic tool for observational studies, it is recognized that a composite score may not fully capture domain-specific biases—particularly confounding by indication in treatment analyses—and that this limitation should be considered when interpreting the GRADE certainty ratings. Disagreements were resolved by consensus. To assess inter-rater reliability, 15 studies were randomly selected, and agreement for NOS items and total scores was evaluated using Cohen’s kappa and the intraclass correlation coefficient.

### 2.4. Data Analysis

Analyses were conducted using Review Manager 5.4 and R 4.4.3. Molecular outcomes were pooled as ORs with 95% CIs, whereas treatment and prognostic outcomes were pooled as adjusted or design-adjusted HRs with 95% CIs. Heterogeneity was evaluated by employing Cochran’s Q test and the I^2^ statistic. Given the anticipated clinical and methodological heterogeneity, random-effects models were used throughout. DerSimonian–Laird estimation was used for the primary analyses, except for outcomes containing five or fewer studies, for which REML estimation was applied. All pooled estimates were further recalculated using restricted maximum likelihood (REML) estimation with a Knapp–Hartung adjustment to derive more conservative 95% confidence intervals (CIs). Furthermore, 95% prediction intervals (PIs) were computed for outcomes with substantial heterogeneity (I^2^ > 50%) or fewer than ten studies, to illustrate the range of true effects expected in future settings. When multiple effect estimates originated from the same study population, they were first combined within the study using an inverse-variance fixed-effect model. For molecular outcomes, zero-event studies were handled using the Mantel–Haenszel method, which accommodates single-zero studies without continuity correction; no double-zero studies were identified in the present dataset. To explore potentially coordinated molecular patterns, post hoc analyses were restricted to studies that concurrently reported p53 protein overexpression and MSI/dMMR, or E-cadherin loss and HER2 positivity. Within each study set, marker-specific ORs were synthesized separately. REML estimation with Knapp–Hartung adjustment was applied when at least two studies were available; otherwise, study-specific estimates were reported descriptively.

Age cutoff was prespecified in PROSPERO as a subgroup variable for the assessment of between-study heterogeneity. Additional post hoc subgroup analyses, including those by geographic region, detection method, and study population, were conducted based on data availability to further investigate potential sources of heterogeneity. Subgroup differences were assessed using formal interaction tests, with a *p*-value for interaction of <0.05 indicating a statistically significant difference. Because individual participant data were unavailable, patients could not be reclassified according to a common age threshold; therefore, the primary analyses synthesized the contrasts reported under each study’s original EOGC definition. Cutoff-stratified subgroup analyses were conducted for each outcome to assess whether the pooled estimates differed according to the study-level EOGC definition. Studies were categorized into two comparison groups based on the age ceiling of the enrolled EOGC cohort: a ≤50 years group, which included studies explicitly defining EOGC as ≤50 years or ≤45 years (as all patients were inherently aged ≤50), and a lower cutoff group, which comprised studies adopting a stricter age threshold of ≤40 years or less (e.g., ≤30 years). Differences between cutoff categories were evaluated using formal tests for subgroup interaction. For overall survival, meta-regression additionally incorporated the age cutoff as a covariate to quantify its contribution to between-study heterogeneity. Robustness was assessed through sensitivity analyses using alternative estimators, leave-one-out exclusion, exclusion of studies with NOS scores ≤6, removal of correlated within-study estimates, and restriction of overlapping database populations. To evaluate the potential influence of participant overlap from the same large database (principally the Surveillance, Epidemiology, and End Results (SEER) program), a sensitivity analysis was conducted for outcomes in which multiple included studies drew on SEER data with overlapping recruitment periods. In this analysis, all but one SEER-based study per outcome—the study with the most recent publication year and the largest sample size—were excluded, and the resulting pooled estimate was compared with that of the main analysis. For outcomes comprising more than ten studies and showing substantial heterogeneity, exploratory univariate and multivariable REML meta-regression was used to examine study-level contributors to heterogeneity. Exploratory univariable REML meta-regression for OS examined age cutoff, region, publication year, sample size, surgical resection rate, chemotherapy use, and the proportion of patients with stage III–IV disease. Multivariable models included age cutoff, region, and standardized sample size; a separate model incorporating surgical resection rate, chemotherapy use, and stage III–IV disease proportion was fitted to the 10 studies reporting all three covariates. For outcomes with more than ten independent estimates, small-study effects were assessed using funnel plots and Egger’s test; potential publication bias was considered when visual asymmetry was accompanied by *p* < 0.05. The certainty of evidence for each clinically important outcome was independently assessed by two reviewers using the GRADE (Grading of Recommendations, Assessment, Development, and Evaluations) framework across five domains—risk of bias, inconsistency, indirectness, imprecision, and publication bias—informed by Newcastle–Ottawa Scale scores, heterogeneity statistics (I^2^ and Cochran’s Q), 95% confidence intervals, and sensitivity/subgroup analyses. Because all included studies were observational, certainty was initially rated as low and was downgraded for serious or very-serious domain-specific limitations or upgraded according to established GRADE criteria. A Summary of Findings table, constructed per GRADE guidelines, reports certainty ratings for the main treatment and prognostic outcomes, with outcome-specific judgments detailed in Table S8.

## 3. Results

### 3.1. Included Studies

**Figure 1:**
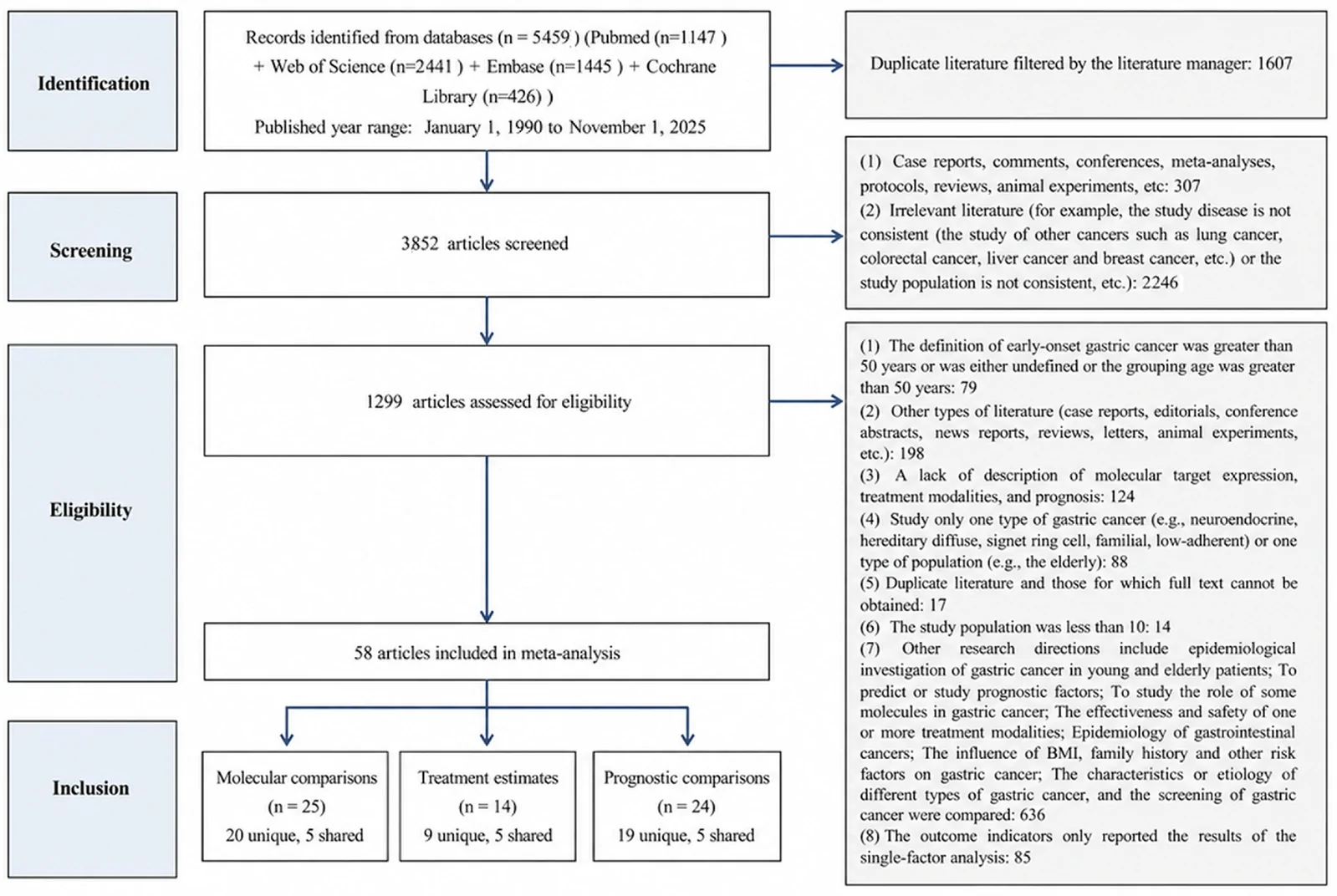
PRISMA flow diagram of study selection.

The database search identified 5459 records across PubMed, Embase, the Web of Science Core Collection, and the Cochrane Library. After duplicates were removed, 3852 records remained. Title and abstract screening excluded 2246 irrelevant records and 307 non-original publications, leaving 1299 reports for full-text assessment. Ultimately, 58 unique studies were included in the quantitative synthesis of molecular characteristics, treatment–survival associations, and prognostic outcomes. The study-selection process is summarized in Figure 1. During full-text assessment, the category “other research directions” (*n* = 636) encompassed studies of non-gastric malignancies, basic mechanisms, genetic syndromes without population-level screening objectives, and treatment outcomes not stratified by age at onset; a detailed breakdown is provided in the Supplementary Note. Study-level distributions of publication period, sample size, design, data source, geographic region, EOGC age cutoff, contributed outcomes, and study quality are summarized in Table 1. Detailed characteristics—including the definitions of EOGC and LOGC, country, design, sample size, molecular detection methods, molecular event frequencies, and prognostic hazard ratios—are provided for each study in Table S2. Of the 58 unique studies, 25 contributed molecular comparisons, 14 contributed treatment–survival association estimates, and 24 contributed prognostic comparisons. Table 2 integrates the pooled estimates with the corresponding robustness assessments for the principal molecular, treatment-associated, and prognostic outcomes. Supplementary estimates recalculated using REML with Knapp–Hartung adjustment are presented in Table S10; the corresponding 95% prediction intervals are provided in Table S7.

The methodological quality of the included studies was assessed using the Newcastle–Ottawa Scale (NOS). Scores ranged from 5 to 9 (mean, 7.26), with 49 studies classified as high quality. Inter-rater agreement was moderate to perfect at the item level (κ = 0.53–1.00), with high overall agreement (κ = 0.862) and excellent reliability for total scores (ICC = 0.892; Tables S3 and S4).

### 3.2. Molecular Characteristics

#### 3.2.1. HER2

**Figure 2:**
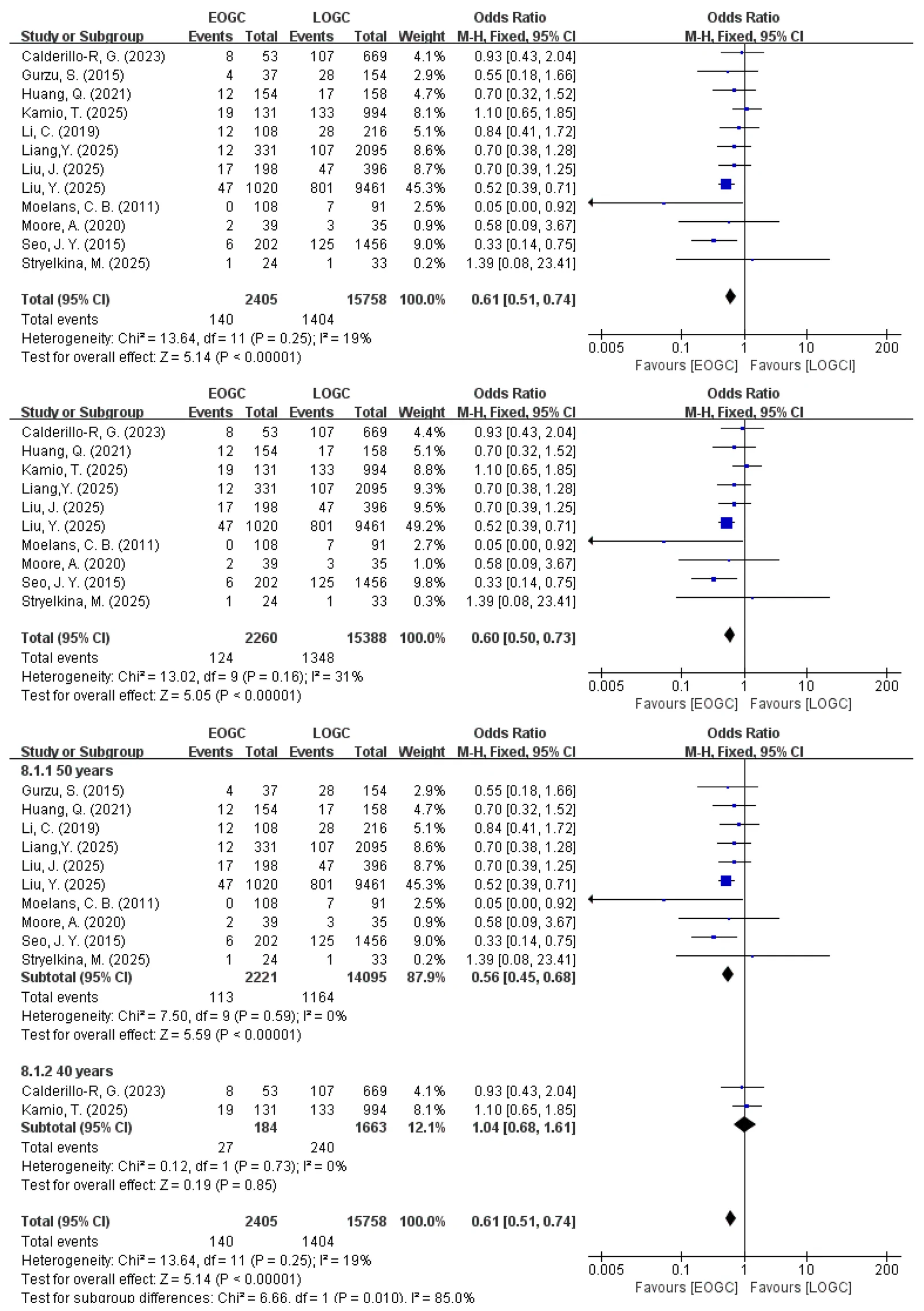
Forest plots for HER2 positivity in EOGC versus LOGC. Forest plots of pooled odds ratios for HER2 positivity in early-onset versus late-onset gastric cancer. Panels show the primary analysis, sensitivity analysis excluding low-quality studies, and subgroup analysis by EOGC age threshold. Studies included in the forest plots: [4,8,9,10,11,13,15,25,26,27,28,29].

Across 12 studies, the primary random-effects meta-analysis showed lower odds of HER2 positivity in EOGC than in LOGC (OR, 0.66; 95% CI, 0.53–0.84; I^2^ = 19%). The effect direction was preserved under REML estimation with Knapp–Hartung adjustment, although the confidence interval crossed the null (OR, 0.74; 95% CI, 0.54–1.01). The estimate was otherwise robust to leave-one-out analysis and exclusion of low-quality studies. Exploratory subgroup analysis indicated variation in the HER2 association according to the EOGC age threshold (>40 to ≤50 years versus ≤40 years; Pinteraction = 0.010). Overall, sensitivity, and age-threshold subgroup estimates are presented in Figure 2.

#### 3.2.2. p53 Protein Overexpression and TP53 Mutation

Two biologically distinct alterations were analyzed separately: p53 protein overexpression assessed by immunohistochemistry (eight studies) and TP53 mutations detected by sequencing (four studies). p53 protein overexpression was less frequent in EOGC than in LOGC (OR, 0.69; 95% CI, 0.60–0.80; I^2^ = 42%), with a consistent estimate obtained using REML (OR, 0.74; 95% CI, 0.59–0.93). The association was retained under the fixed-effect model and leave-one-out analyses but was attenuated after low-quality studies were excluded, indicating only moderate robustness. The pooled estimate did not vary according to the definition of p53 protein positivity; however, an exploratory interaction with the EOGC age threshold was observed (Pinteraction = 0.020). In the REML analysis, the pooled estimate favored fewer TP53 mutations in EOGC, but was imprecise and compatible with no association (OR, 0.60; 95% CI, 0.28–1.29; I^2^ = 25%). Exclusion of Dhobi et al. 2013 [23] materially altered the pooled estimate, indicating dependence on individual studies.

#### 3.2.3. MSI/dMMR

Ten studies reported MSI/dMMR status. The random-effects model showed lower odds of MSI/dMMR positivity in EOGC than in LOGC, accompanied by substantial heterogeneity (OR, 0.33; 95% CI, 0.12–0.91; I^2^ = 68%). The effect direction was maintained in fixed-effect and study-quality sensitivity analyses; however, the confidence interval crossed the null when Moore et al. 2020 [29], Liu et al. 2023 [10], or Seo et al. 2015 [11] was individually excluded, indicating sensitivity to individual studies. Subgroup analyses by assay, geographic region, and EOGC age threshold did not explain the observed heterogeneity.

#### 3.2.4. EBV (EBV Infection Status)

Random-effects meta-analysis showed no statistically significant difference in EBV positivity between EOGC and LOGC; the REML estimate was similarly nonsignificant but imprecise (OR, 1.19; 95% CI, 0.41–3.49; I^2^ = 68%). The result remained nonsignificant under a fixed-effect model, leave-one-out analysis, and exclusion of low-quality studies. Subgroup analyses stratified by detection method and age cutoff likewise identified no statistically significant between-group differences. Given the small evidence base and substantial heterogeneity, these analyses indicate no detectable association with age group rather than evidence of equivalent EBV prevalence.

#### 3.2.5. E-Cadherin

Six studies evaluated loss of E-cadherin expression. The random-effects model indicated no significant overall distinction between EOGC and LOGC (OR, 2.23; 95% CI, 0.86–5.76), with very high heterogeneity (I^2^ = 89%). The instability of the pooled estimate was reinforced by both the REML analysis (OR, 3.33; 95% CI, 0.20–54.93) and the 95% prediction interval (0.004–2626.422). After exclusion of one outlying study [30], the prediction interval narrowed to 0.182–9.505 but continued to encompass substantial uncertainty, while the pooled estimate became more stable (Table S7). Sensitivity analyses demonstrated model- and study-level instability: statistical significance was reached under the fixed-effect model and after exclusion of Silva et al. [31], but not after exclusion of low-quality studies. Exclusion of the outlying study preserved the direction of association but yielded a nonsignificant estimate (OR, 1.32; 95% CI, 0.68–2.56). These findings indicate that the overall E-cadherin estimate should be interpreted cautiously, but that standardized immunohistochemistry (IHC) interpretation criteria may identify a more consistent signal. Restriction to studies defining E-cadherin loss uniformly as membranous immunoreactivity in <10% of tumor cells markedly reduced heterogeneity (I^2^ = 13%) and yielded a significant association (OR, 1.82; 95% CI, 1.16–2.85). In contrast, studies using other criteria remained highly heterogeneous. The substantial heterogeneity in the primary analysis therefore appears to have been driven, at least partly, by variation in IHC scoring criteria. This exploratory finding suggests that E-cadherin loss may be more frequent in EOGC and identifies assay harmonization as a prerequisite for prospective validation.

#### 3.2.6. Analyses Restricted to Co-Reporting Studies

Three studies (Gurzu et al. 2015 [13], Lim et al. 2003 [32], and Seo et al. 2015 [11]) reported both p53 protein overexpression and MSI/dMMR. In this common study set, MSI/dMMR was significantly less frequent in EOGC (REML–Knapp–Hartung OR, 0.127; 95% CI, 0.042–0.391; I^2^ = 0%), whereas p53 protein overexpression did not differ significantly between EOGC and LOGC (OR, 0.733; 95% CI, 0.356–1.512; I^2^ = 67.7%).

Only Gurzu et al. 2015 [13] reported both E-cadherin and HER2. E-cadherin loss was more frequent in EOGC (OR, 2.63; 95% CI, 1.10–6.28), whereas HER2 positivity was not significantly different (OR, 0.55; 95% CI, 0.18–1.66). Because only one study was available and cross-classified biomarker data were not reported, no joint meta-analysis was performed.

### 3.3. Treatment

#### 3.3.1. Radical Resection

**Figure 3:**
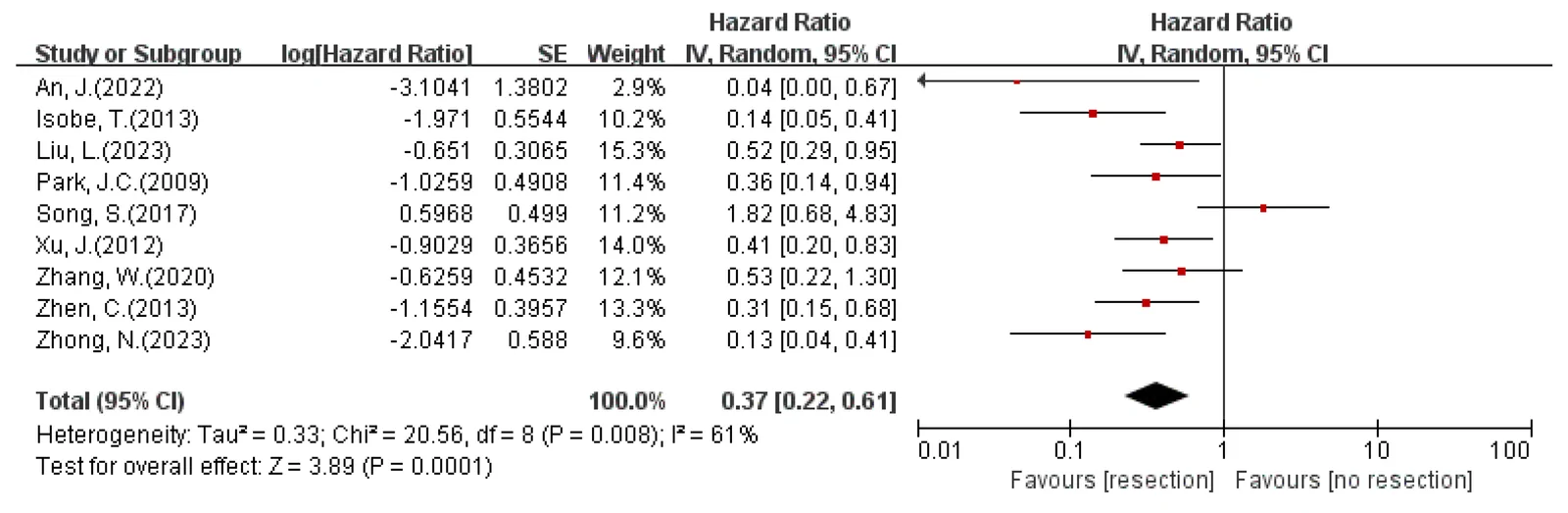

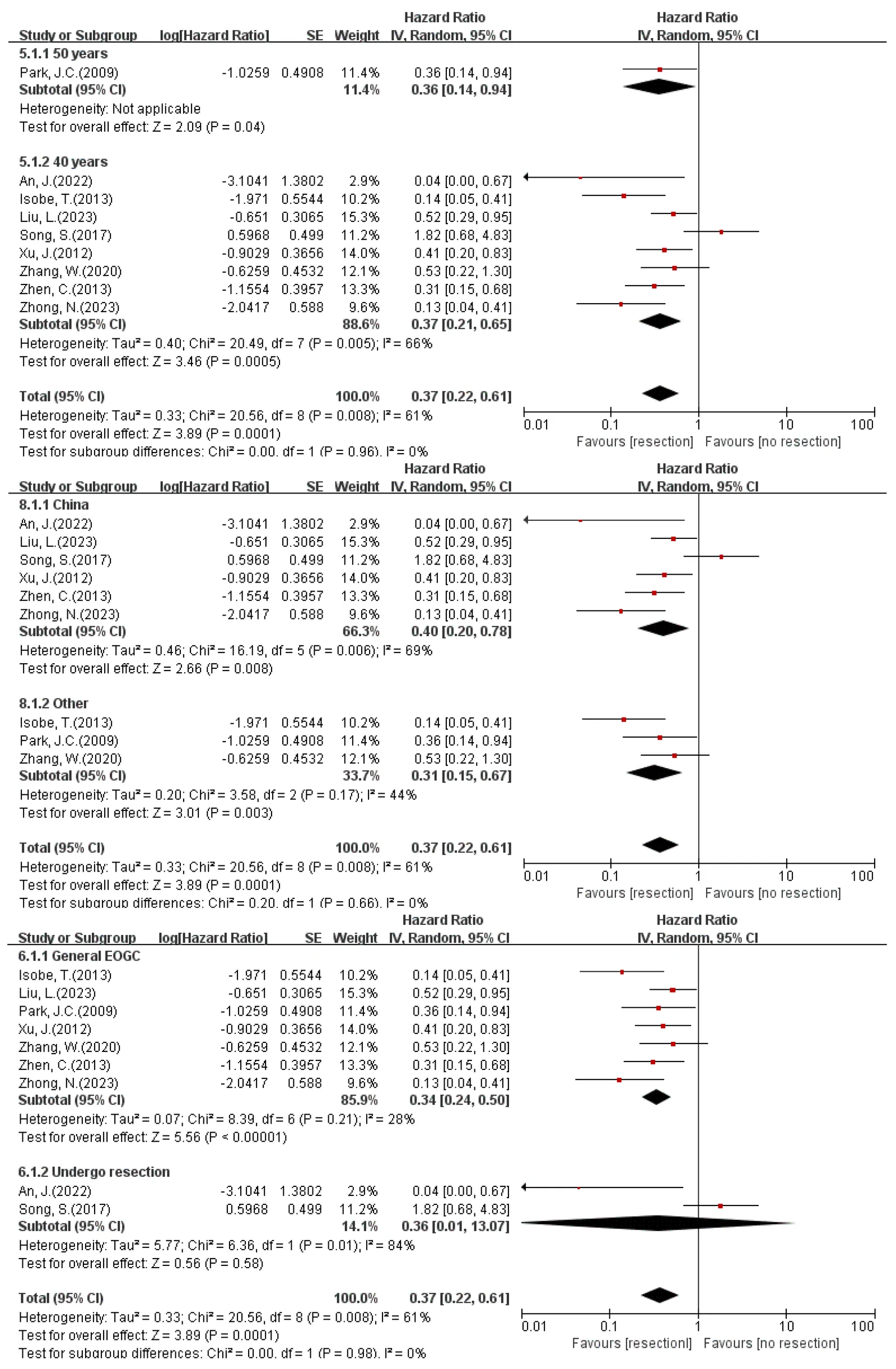
Forest plots for the survival association of radical resection in EOGC. Forest plots evaluate the association between radical resection and overall survival among patients with early-onset gastric cancer. Panels show the primary pooled analysis and subgroup analyses stratified by age cutoff, geographic region, and study population. Studies included in the forest plots: [33,34,35,36,37,38,39,40,41].

Nine observational studies examined the association between radical resection and overall survival among patients with EOGC. Given the potential for confounding by indication and treatment-selection bias, this association should not be interpreted as evidence of a causal survival benefit. In the primary random-effects analysis, radical resection was associated with lower mortality (HR, 0.37; 95% CI, 0.22–0.61), with moderate between-study heterogeneity (I^2^ = 61%). After REML estimation with Knapp–Hartung adjustment, the confidence interval remained below the null (95% CI, 0.189–0.716; Table S7), supporting the pooled mean association. However, the 95% prediction interval crossed the null (0.076–1.772), indicating that the magnitude and direction of the association may vary across comparable clinical settings. The association was retained in the fixed-effect, leave-one-out, and analyses excluding low-quality studies, indicating robustness of the pooled estimate to these analytical assumptions. No evidence of effect modification was detected across subgroups defined by age cutoff, geographic region, or study population. The main and subgroup analyses are shown in Figure 3.

#### 3.3.2. Chemotherapy

Five observational studies examined the association between chemotherapy and overall survival among patients with EOGC. In the primary random-effects analysis, receipt of chemotherapy was associated with lower observed mortality (HR, 0.69; 95% CI, 0.57–0.84), with no between-study heterogeneity detected (I^2^ = 0%). However, REML estimation with Knapp–Hartung adjustment yielded a less precise estimate whose confidence interval crossed the null (HR, 0.73; 95% CI, 0.52–1.02), weakening confidence in the association. The corresponding 95% prediction interval also crossed the null (0.448–1.191; Table S7), indicating that the underlying association may vary across comparable populations and treatment settings. The estimate was similar under a fixed-effect model and after exclusion of one reconstructed HR and one pooled HR (HR, 0.66; 95% CI, 0.53–0.82). Exclusion of the study contributing a correlated estimate likewise produced a materially unchanged result (HR, 0.67; 95% CI, 0.55–0.83; I^2^ = 0%). Nevertheless, statistical significance was lost after exclusion of An et al. 2023 [24], indicating that the pooled association depended partly on this single study and was therefore not robust. Because this study was restricted to patients with metastatic disease, differences in stage distribution and treatment indication may have contributed to the observed association. No evidence of effect modification was detected by patient type or age cutoff. Given the influence of a single metastatic-disease cohort and incomplete adjustment for TNM stage, the association should be considered exploratory and cannot support a generalized chemotherapy recommendation based on early-onset status alone.

### 3.4. Prognosis

#### 3.4.1. OS

**Figure 4:**
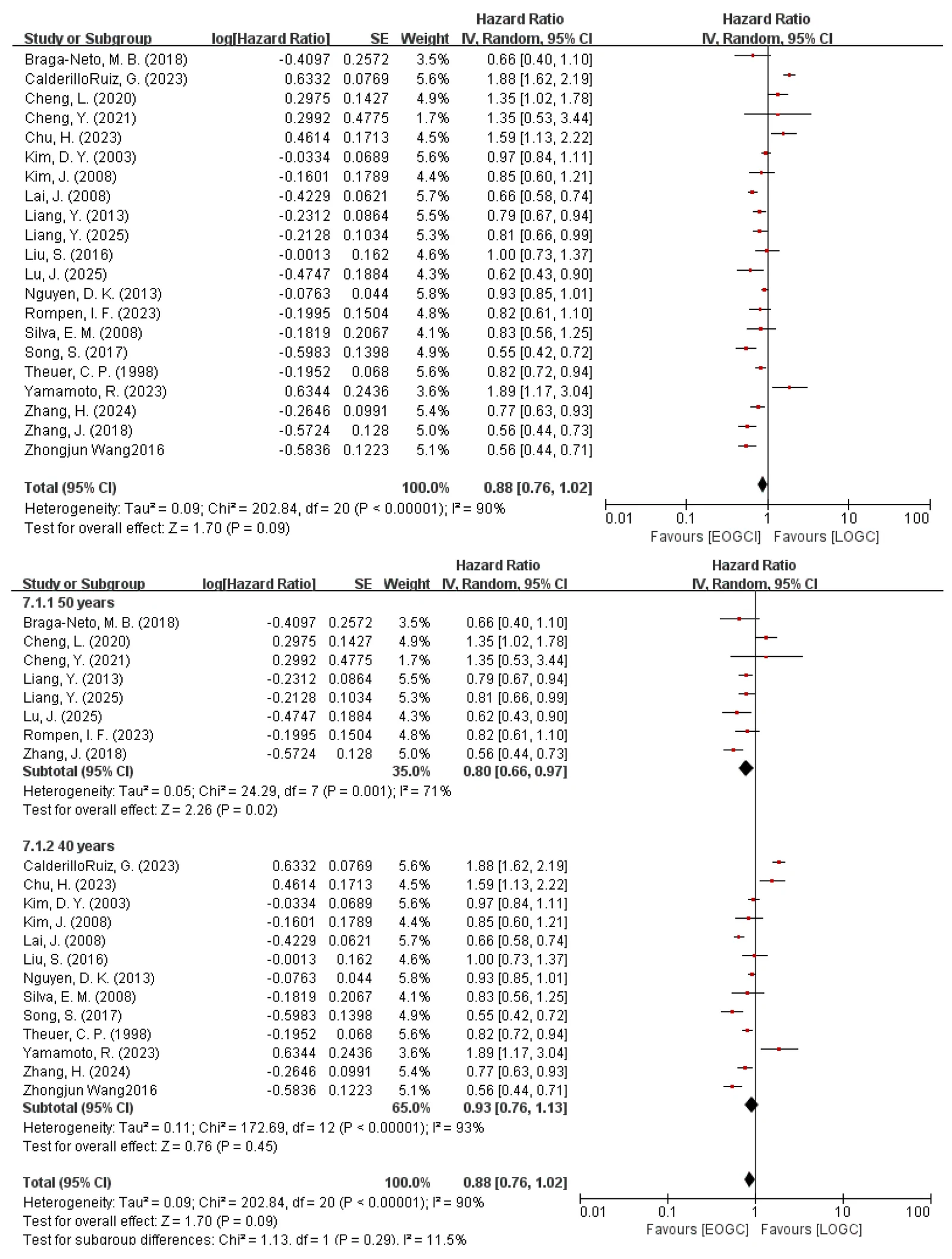

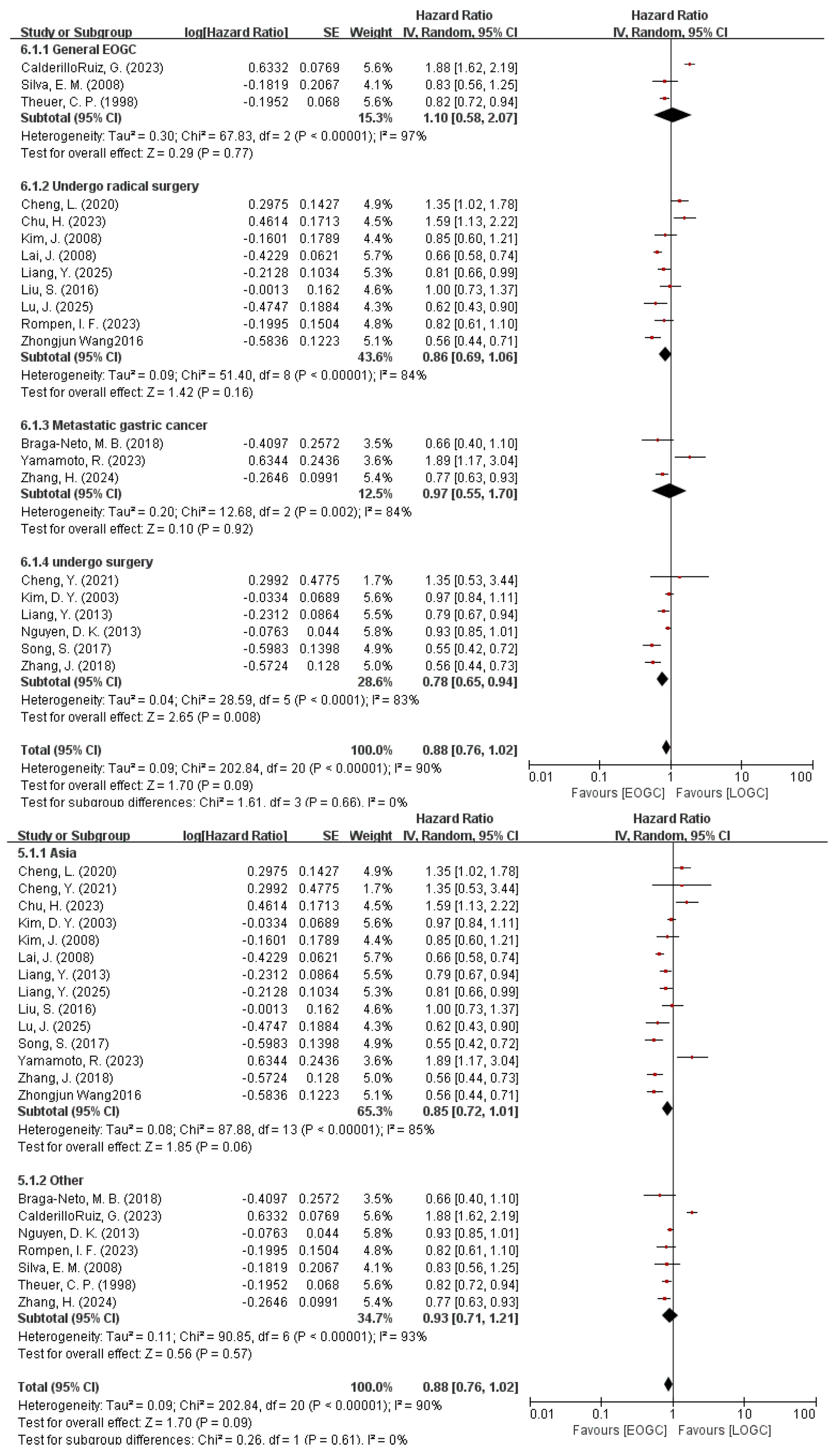
Forest plots for overall survival in EOGC versus LOGC [6,15,16,17,18,25,31,37,42,43,44,45,46,47,48,49,50,51,52,53,54].

Twenty-one studies evaluated OS differences between EOGC and LOGC. The random-effects model yielded a pooled HR of 0.88 (95% CI, 0.76–1.02), with substantial between-study heterogeneity (I^2^ = 90%). The 95% prediction interval (0.429–1.811; Table S7) encompassed clinically meaningful effects in either direction, indicating that no consistent survival advantage could be inferred across comparable clinical settings. Sensitivity analyses demonstrated that the estimated association was sensitive to model specification and the influence of individual studies. Under a fixed-effect model, a modest OS advantage was observed for EOGC (HR, 0.88; 95% CI, 0.85–0.92). Exclusion of Calderillo-Ruíz et al., 2023 [25] strengthened this association (HR, 0.83; 95% CI, 0.74–0.93). By contrast, exclusion of reconstructed HRs and restriction to high-quality studies produced estimates consistent with the primary analysis, although both confidence intervals included unity (HR, 0.91; 95% CI, 0.74–1.12 and HR, 0.84; 95% CI, 0.70–1.02, respectively). No significant effect modification was detected by age cutoff or geographic region, whereas the subgroup restricted to surgically treated patients showed better OS in EOGC. This apparent advantage may reflect selection for resectable disease and favorable performance status rather than an age-related biological effect and should therefore be interpreted as exploratory. To address potential population overlap, the two earlier SEER-based studies were excluded while the most recent report was retained; the pooled estimate remained materially unchanged (HR, 0.90; 95% CI, 0.76–1.07; I^2^ = 90%). Similarly, exclusion of four studies containing EOGC subgroups that shared a common LOGC reference yielded an HR of 0.91 (95% CI, 0.74–1.12; I^2^ = 91%), showing that potentially correlated estimates did not account for the observed uncertainty or heterogeneity. The main and subgroup analyses are shown in Figure 4.

Forest plots compare overall survival between early-onset and late-onset gastric cancer. For four studies reporting multiple eligible comparisons, within-study HRs were first combined using fixed-effect models to obtain independent study-level estimates. Panels present the primary meta-analysis and subgroup analyses according to age cutoff, geographic region, and treatment background.

Exploratory meta-regression identified no significant association between the OS effect estimate and age cutoff, region, publication year, or sample size. Surgical resection rate (*p* = 0.450; R^2^ = 0%), chemotherapy use (*p* = 0.553; R^2^ = 0%), and the proportion of patients with stage III–IV disease (*p* = 0.335; R^2^ = 0.5%) were likewise non-significant in univariate models. Among the 10 studies reporting all three covariates, the multivariable model did not significantly explain between-study heterogeneity (QM *p* = 0.874), and substantial residual heterogeneity remained (I^2^ = 88.9%; Table S5). These null moderator tests should not be interpreted as evidence that treatment and stage do not influence survival, given the limited number of studies, study-level aggregation, and risks of low power and model overfitting. Reported treatment patterns are summarized in Table S6. Studies included in the forest plots: [6,15,16,17,18,25,31,37,42,43,44,45,46,47,48,49,50,51,52,53,54].

#### 3.4.2. DFS

Five studies compared DFS between EOGC and LOGC. Using the REML estimator, no significant difference in DFS was observed between EOGC and LOGC (HR, 1.24; 95% CI, 0.82–1.88; I^2^ = 57%). The 95% prediction interval spanned potential benefit and harm (0.539–2.839; Table S7), indicating substantial uncertainty in the effect expected across clinical settings. The statistical significance of the DFS estimate was sensitive to both model specification and individual studies: the fixed-effect model yielded an HR of 1.24 (95% CI, 1.03–1.49), whereas exclusion of Ramos et al. [55] yielded an HR of 1.37 (95% CI, 1.05–1.79). No significant subgroup difference was identified by age cutoff. By geographic setting, however, EOGC was associated with poorer DFS in Chinese cohorts (HR, 1.61; 95% CI, 1.25–2.09; *p* for interaction = 0.008). All three Chinese studies [18,43,50] were single-center retrospective cohorts with potential selection bias and heterogeneous follow-up and adjuvant treatment; this subgroup finding represents a cohort-specific signal rather than nationally generalizable evidence.

#### 3.4.3. CSS

Four studies compared CSS between EOGC and LOGC. The REML analysis showed no significant difference in CSS between EOGC and LOGC (HR, 0.87; 95% CI, 0.65–1.15), with considerable between-study heterogeneity (I^2^ = 92%). The corresponding 95% prediction interval was 0.473–1.586 (Table S7), indicating that the direction of association may vary across settings. The apparent CSS advantage for EOGC emerged only in sensitivity analyses: exclusion of Song et al., 2016 [56] (HR, 0.84; 95% CI, 0.81–0.87), use of a fixed-effect model (HR, 0.88; 95% CI, 0.85–0.91), and exclusion of the reconstructed HR (HR, 0.75; 95% CI, 0.67–0.83; I^2^ = 18%) all yielded statistically significant estimates. Collectively, these analyses suggest a possible CSS advantage for EOGC when non-cancer deaths are excluded from the endpoint, although this association was not robust to analytical specification. After studies with shared comparators were excluded, only two studies remained (HR, 0.75; 95% CI, 0.66–0.85; I^2^ = 18%), substantially limiting the reliability and generalizability of this estimate.

### 3.5. Assessment of Publication Bias

**Figure 5:**
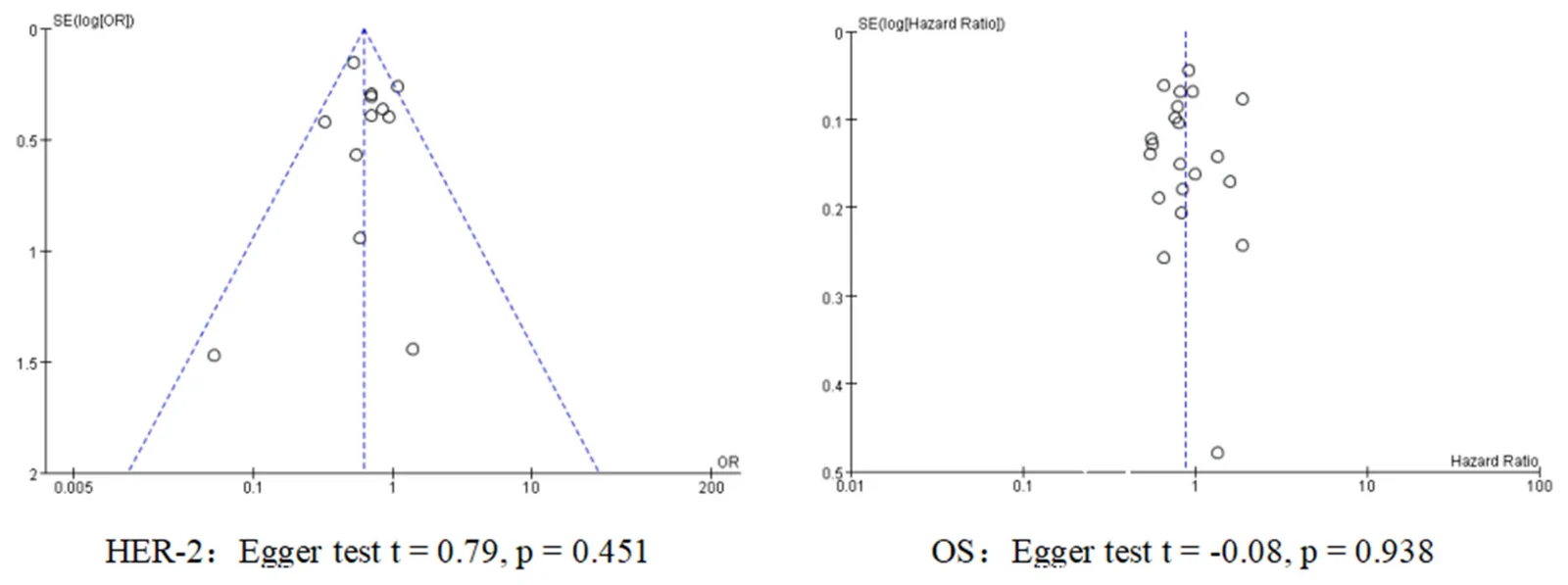
The funnel plot and Egger test value of HER2 and OS.

Publication bias and small-study effects were assessed for HER2 positivity and OS, the only outcomes represented by at least 10 independent estimates. Funnel plots were approximately symmetric, and Egger’s regression test provided no evidence of small-study effects for either outcome (HER2: t = 0.79, *p* = 0.451; OS: t = −0.08, *p* = 0.938; Figure 5). Interpretation of the OS funnel plot was nevertheless limited by the very high between-study heterogeneity. Formal assessment was not performed for the remaining molecular markers—p53 overexpression, TP53 mutation, MSI/dMMR, EBV positivity, and E-cadherin loss—or for other outcomes supported by fewer than 10 studies, precluding reliable evaluation of small-study effects. Any observed funnel-plot asymmetry may arise from genuine heterogeneity, methodological differences, or other mechanisms unrelated to publication bias. Accordingly, these analyses were considered exploratory and should not be interpreted as establishing the absence of publication bias.

### 3.6. Certainty of Evidence Assessment

The certainty of evidence across all outcomes was evaluated using the GRADE framework and is summarized in Table S8. Certainty was rated low for HER2 positivity, p53 overexpression, TP53 mutation, MSI/dMMR, and EBV positivity, but very low for E-cadherin loss in the overall analysis, owing primarily to the observational evidence base and moderate to very-serious inconsistency, with additional imprecision for E-cadherin loss. For treatment outcomes, certainty was very low for both radical resection and chemotherapy: the association between radical resection and lower mortality was limited by very-serious risk of bias and serious inconsistency, whereas the chemotherapy estimate was unstable under leave-one-out sensitivity analysis. Certainty was uniformly very low for OS, DFS, and CSS, driven primarily by very-serious inconsistency, with additional concerns regarding risk of bias and imprecision for DFS. Collectively, these assessments identify the outcomes most vulnerable to bias, inconsistency, and imprecision, thereby distinguishing relatively stable findings from associations that remain hypothesis-generating.

## 4. Discussion

### 4.1. Core Findings

This systematic review and meta-analysis synthesized molecular, therapeutic, and prognostic evidence comparing EOGC and LOGC, while examining how biomarker definitions, study settings, and analytical decisions influenced the pooled estimates. EOGC showed lower pooled prevalences of HER2 positivity, MSI/dMMR positivity, p53 protein overexpression, and TP53 mutations, whereas greater E-cadherin loss emerged only in an assay-restricted analysis. The HER2 and p53 estimates varied across study-level cutoff categories, indicating that these associations were assay-dependent and should not be interpreted as threshold-independent biological differences. Among the treatment-related factors evaluated in EOGC, radical resection showed the most pronounced association with lower mortality, whereas the association between chemotherapy and survival was not robust across analyses. The available evidence did not establish a consistent difference in OS, DFS, or CSS between EOGC and LOGC. A signal of shorter DFS was largely confined to Chinese single-center cohorts, whereas sensitivity analysis suggested a possible CSS advantage. Both signals were based on few studies and accompanied by substantial heterogeneity, precluding firm prognostic conclusions. Because most pooled odds ratios were derived from unadjusted comparisons, the findings should be interpreted as weighted between-group prevalence patterns rather than independent age-specific effects.

### 4.2. Comparison with Previous Evidence and Interpretation of Results

#### 4.2.1. Molecular Characteristics

HER2: The lower HER2 positivity in EOGC is consistent with the meta-analysis by Lin et al. [20]. Of note, this was published online ahead of print after the closure of our systematic search (November 2025) and was therefore not included in the quantitative synthesis; it is cited here solely as a discussion comparator given its direct relevance. An updated search will be conducted before the manuscript is published to ensure that the quantitative synthesis remains current. HER2 positivity is more frequently observed in intestinal-type and proximal/gastroesophageal junction tumors and has also been associated with male sex [57,58]. In contrast, EOGC occurs more frequently in women and is enriched for diffuse-type and non-cardia disease [7]. Therefore, the lower observed HER2 positivity in EOGC may partly reflect differences in sex, histologic subtype, and tumor location rather than an independent age-related effect. Intratumoral HER2 heterogeneity may further contribute to variation in detection [59].

p53 overexpression and TP53 mutations: The lower prevalence of p53 protein overexpression and TP53 mutations in EOGC may partly reflect its histologic composition. Diffuse-type tumors may harbor truncating, frameshift, or other loss-of-function TP53 variants that produce little or no p53 protein accumulation on IHC [60]. By contrast, intestinal-type tumors, which are more common in LOGC, more frequently harbor missense TP53 variants that can stabilize mutant p53 protein and produce nuclear accumulation detectable by IHC [60]. Although Helicobacter pylori infection may promote p53 degradation through the USF1 pathway [61], the observed higher p53 positivity in LOGC may be partly attributable to missense mutation-driven p53 accumulation. Nevertheless, the pooled estimate for p53 protein overexpression remains sensitive to heterogeneous IHC positivity thresholds, underscoring that protein expression and genomic TP53 mutations are not interchangeable endpoints.

MSI/dMMR: The lower MSI/dMMR positivity in EOGC was consistent with Lin et al. [20] and may be attributable to the biology of MSI. MSI commonly arises from mismatch repair deficiency caused by epigenetic silencing of MLH1 through promoter hypermethylation, an age-associated event [62]. MSI is also more prevalent in intestinal-type than in diffuse-type gastric cancer [63,64]. The younger age and greater prevalence of diffuse-type histology in EOGC may therefore contribute to its lower MSI/dMMR prevalence, although study-level data cannot establish mediation by either factor. However, the result was sensitive to Moore et al., 2020 [29], Liu et al., 2023 [10], and Seo et al., 2015 [11], which documented extremely low MSI-positive rates in EOGC from high-incidence regions. These sparse events contributed to estimate instability and limited the geographical generalizability of the finding.

E-cadherin: In the primary analysis, E-cadherin loss did not differ significantly between EOGC and LOGC, consistent with previous evidence [20]. However, when the analysis was restricted to studies defining E-cadherin loss as staining in <10% of tumor cells, loss was more frequent in EOGC. This discrepancy may reflect variation in IHC scoring systems, including higher positivity thresholds or classification of weak staining as positive. As a core component of adherens junctions, E-cadherin maintains epithelial cohesion; its loss may therefore facilitate discohesive growth and invasion [65]. The divergence between the primary and assay-restricted analyses indicates that standardized E-cadherin scoring is necessary for valid age-group comparisons. Although this exploratory signal is biologically compatible with the role of CDH1 in hereditary diffuse gastric cancer, germline CDH1 and CTNNA1 status was not evaluated; no genetic-testing implications can therefore be inferred from the present meta-analysis.

Integrated analyses of co-reported biomarkers: Recent gastric cancer studies have evaluated MMR, p53, E-cadherin, and HER2 within integrated molecular frameworks while retaining them as distinct biomarkers [66,67]. In our restricted analysis, MSI/dMMR remained less frequent in EOGC, whereas p53 protein overexpression did not differ between groups. The divergent E-cadherin and HER2 estimates reported by Gurzu et al. [13] may similarly reflect histologic or molecular subtype composition rather than coordinated alterations. These findings support joint evaluation but not combination into a single effect estimate.

EBV: The absence of a significant difference in EBV positivity between EOGC and LOGC is consistent with previous reports [20]. EBV-associated gastric cancer more commonly affects middle-aged or older men, often arises in the upper or middle stomach, and is frequently characterized by lymphoid stroma [68]. In contrast, EOGC is more often non-cardia, diffuse-type, or poorly differentiated disease, with molecular drivers more closely related to CDH1 inactivation than EBV-associated hypermethylation or PIK3CA mutations [64]. These differences may explain the absence of clear EBV enrichment in EOGC.

#### 4.2.2. Treatment-Associated Survival

Treatment analyses compared treated and untreated patients within EOGC cohorts and therefore cannot determine whether treatment-associated survival differs between EOGC and LOGC.

Radical resection: Although EOGC frequently exhibits aggressive clinicopathologic features [7], the consistently strong association observed for radical resection—which persisted across sensitivity analyses—may be partially explained by the better nutritional status, fewer comorbidities, and greater postoperative recovery capacity of younger patients [69]. Such physiological reserve may facilitate extended lymphadenectomy or multivisceral resection and improve the completion of postoperative therapy, potentially contributing to higher R0 resection rates [17]. However, confounding by indication and surgical candidacy remains likely, because patients selected for curative surgery generally have more favorable resectability and physiological profiles. These findings therefore support consideration of curative-intent resection in eligible patients and argue against withholding surgery solely because of young age or locally advanced disease [24]; however, they do not establish an independent causal survival benefit.

Chemotherapy: The unstable association between chemotherapy and survival may reflect heterogeneity in molecular subtype, disease stage, and treatment background. Lower frequencies of HER2 positivity and MSI/dMMR may reduce eligibility for biomarker-directed therapies [28,70], whereas the lower TP53 mutation frequency reported in EOGC could theoretically preserve p53-mediated DNA-damage responses and apoptosis, thereby increasing sensitivity to DNA-damaging agents [71]. This mechanistic hypothesis, however, has not been tested directly in EOGC cohorts. The pooled association was attenuated to non-significance after exclusion of An et al., 2023 [24], a cohort restricted to metastatic EOGC, indicating that differences in stage distribution and treatment selection may have disproportionately influenced the summary estimate. Current evidence therefore does not support a uniform chemotherapy–survival association across EOGC populations, underscoring the need for stage-stratified, molecularly annotated prospective studies.

#### 4.2.3. Prognosis

OS: The pooled OS association remained inconclusive because of marked between-study heterogeneity (I^2^ = 90%) and a wide 95% prediction interval encompassing associations in either direction (0.429–1.811), despite its consistency with the non-significant summary reported by Li et al. [7]. This heterogeneity may reflect competing determinants: lower non-cancer mortality [72] may favor OS in EOGC, whereas advanced TNM stage, diffuse-type histology, and poor differentiation [70] may worsen cancer-related outcomes. Aggregate data cannot quantify their relative contributions and therefore cannot establish prognostic equivalence.

DFS: This analysis provides an early quantitative synthesis of DFS differences between EOGC and LOGC. Although the pooled DFS estimate was non-significant, the small evidence base and substantial heterogeneity precluded a reliable inference; the Chinese subgroup nevertheless showed an exploratory signal toward poorer DFS in EOGC. Because the Chinese subgroup evidence was derived exclusively from single-center retrospective cohorts, it remains vulnerable to selection bias and treatment heterogeneity. The finding should therefore be regarded as hypothesis-generating and used to prioritize multicenter validation of recurrence risk in Chinese EOGC populations.

CSS: The primary CSS estimate was inconclusive because only four studies were available, heterogeneity was substantial, and the result was sensitive to both model specification and influential studies. A possible CSS advantage emerged after exclusion of Song et al., 2016 [56]; however, that study compared multiple EOGC age subgroups with a single LOGC reference group, a structure that complicates interpretation and may have contributed to residual heterogeneity despite within-study pooling. Accordingly, the apparent CSS advantage remains tentative and requires confirmation in independent, adequately adjusted cohorts.

Across OS, DFS, and CSS, non-significant summary estimates should not be equated with prognostic equivalence. The pooled HRs represent average associations across clinically heterogeneous populations and cannot define either the direction or magnitude of age-associated survival differences.

By integrating pooled estimates with prediction intervals, model-based sensitivity analyses, and influential-study assessments, this review distinguishes robust findings from unstable signals, prevents overinterpretation of null average effects, and defines the methodological priorities for future studies. Prospective multicenter cohorts should use prespecified age thresholds, harmonized staging and eligibility criteria, standardized outcomes, comparable treatment backgrounds, and uniform adjustment for major prognostic factors.

### 4.3. Clinical Implications

By distinguishing findings that can inform current practice from those that remain hypothesis-generating, this synthesis provides a clinically useful framework for the management of EOGC. Current evidence does not support using chronological age alone to modify guideline-recommended biomarker testing, systemic therapy selection, surgical assessment, or postoperative surveillance.

First, it is hypothesized that molecular testing may help guide treatment selection in EOGC. HER2 positivity and dMMR/MSI-H status are established predictors of benefit from trastuzumab and PD-1 blockade [73,74]. Their lower pooled prevalence in EOGC should not be extrapolated to individual treatment eligibility. Instead, these unadjusted prevalence patterns reinforce that comprehensive biomarker testing should remain the standard for all patients, as a subset of young individuals may still harbor actionable HER2 amplification or MSI-H/dMMR and could derive benefit from matched therapies. The exploratory E-cadherin signal and the reported enrichment of CLDN18–ARHGAP fusions [75] further define priorities for molecular investigation but are not yet suitable for therapeutic stratification.

Second, curative-intent resection was consistently associated with reduced mortality across the included studies. Because this association remains vulnerable to confounding by indication and patient selection, it should be interpreted as being concordant with—rather than independently validating—current surgical practice. Age alone should therefore neither preclude nor mandate surgery; candidacy should be determined through standard multidisciplinary assessment of tumor resectability, disease stage, physiological fitness, and treatment goals.

Third, the effect of conventional chemotherapy in EOGC remains unresolved, and the available evidence does not establish either an age-specific survival benefit or differential treatment sensitivity. For example, Kamio et al. found that the TP53 R175H mutation is more common in EOGC and may be associated with oxaliplatin sensitivity [9]. Prospective trials are needed to evaluate whether EOGC has differential sensitivity to systemic therapies, including platinum-based regimens and other novel agents.

Finally, the signal for poorer DFS, identified in an exploratory subgroup analysis of three Chinese single-center retrospective cohorts, is insufficient to support intensified surveillance. Its generalizability is constrained by the limited evidence base and potential confounding from disease-stage distribution, institutional treatment protocols, and follow-up intensity. Nevertheless, this finding identifies postoperative recurrence as a priority for prospective multicenter investigation and may inform the design of future risk-stratified surveillance studies.

Collectively, this synthesis helps prevent premature age-based treatment escalation or de-escalation while identifying biomarker testing, molecular stratification, and recurrence-risk validation as the most clinically relevant priorities for future research.

### 4.4. Heterogeneity and Limitations

Substantial between-study heterogeneity was observed for several outcomes, potentially reflecting differences in age definitions, biomarker assays, geographic and histological composition, treatment context, and study era. EOGC thresholds ranged from ≤30 to ≤50 years, altering the composition of both the EOGC and LOGC groups and limiting direct comparability across studies. This variability was examined through prespecified cutoff-stratified subgroup analyses, exploratory meta-regression for OS, assay-restricted sensitivity analyses, and 95% prediction intervals (PIs). Although most pooled estimates remained directionally stable, attenuation in some subgroups and persistent residual heterogeneity indicate that the results represent average associations across the definitions examined rather than age-threshold-invariant effects.

For molecular outcomes, heterogeneity likely reflected variation in MSI/dMMR platforms [63], regional differences in EBV-associated gastric cancer, and temporal changes in biomarker assessment. Restriction to studies using uniform immunohistochemical criteria substantially reduced heterogeneity in E-cadherin and p53 overexpression, suggesting that assay and scoring variability contributed to the primary estimates. The cohort collection periods, which extended from 1965 to 2024, also spanned major changes in biomarker testing, staging, and treatment.

Treatment-associated estimates were constrained by clinically heterogeneous comparator groups. For radical resection, the reference groups included patients undergoing non-curative or palliative procedures and those receiving no surgery. The observed mortality association therefore cannot separate a potential treatment effect from patient selection, surgical fitness, and confounding by indication and should not be interpreted as a causal estimate of surgical efficacy.

OS remained highly heterogeneous after subgroup and meta-regression analyses. This is expected because OS is influenced not only by tumor biology but also by non-cancer mortality, baseline comorbidities, treatment tolerance, adherence, and subsequent therapies [8,76]. Lower noncancer mortality among younger patients may partly counterbalance aggressive disease characteristics, contributing to comparable OS between EOGC and LOGC [72]. Across the 21 studies reporting OS, the proportion of patients with stage III–IV disease ranged from approximately 30% to 100%, surgical resection rates ranged from 10.5% to 100%, and chemotherapy use differed by more than tenfold (Table S6). Neither univariable analyses of resection rate, chemotherapy use, and stage III–IV disease proportion nor the corresponding multivariable model materially explained OS heterogeneity; residual I^2^ remained 88.9% in the 10 studies with complete covariate reporting (Table S5). Residual heterogeneity may arise from differences in specific T/N categories, R0 resection status, chemotherapy regimens and duration, and covariate adjustment, which would be more appropriately evaluated through an individual participant data meta-analysis. Differences in recurrence patterns and follow-up practices may similarly contribute to DFS heterogeneity. Standardized reporting of Lauren classification, TNM stage, treatment sequence, margin status, recurrence site, and cause-specific mortality would allow more clinically meaningful synthesis than age cutoff alone.

The 95% PIs further demonstrated the clinical implications of this uncertainty (Table S7). For OS, the PI crossed unity, indicating that the direction of association may differ in a future cohort. The extremely wide PI for E-cadherin (0.004–2626.422) reflected assay heterogeneity and the influence of an outlying study. Future studies should therefore prioritize prespecified age thresholds, harmonized eligibility and stage criteria, comparable treatment backgrounds, standardized biomarker and outcome assessment, and prospective multicenter designs. Consistently, GRADE rated the certainty of evidence as low or very low for most outcomes. Estimates for radical resection, chemotherapy, OS, DFS, and CSS were supported by very low-certainty evidence because of observational study designs, risk of bias, serious inconsistency, sensitivity to individual studies, or imprecision. These findings should therefore be interpreted as hypothesis-generating associations rather than causal or universally generalizable effects.

Several limitations should be acknowledged. First, the majority of included studies were retrospective, and approximately two-thirds originated from Asia, particularly China, South Korea, and Japan. Given the well-documented differences in gastric cancer epidemiology, treatment paradigms, and molecular characteristics—including EBV prevalence—between Asian and Western populations, this geographic concentration substantially limits the generalizability of our findings to non-Asian settings. Second, the absence of a consensus age-based definition of EOGC represented a major source of clinical heterogeneity and limited direct comparability across studies. Although cutoff-stratified subgroup analyses and study-level meta-regression were performed, these approaches could not harmonize individual patients or fully account for changes in the composition of the comparator groups. Age-stratified molecular data were rarely reported according to Lauren classification, tumor location, sex, or stage, and most molecular odds ratios were derived from crude event counts. These estimates therefore describe differences in biomarker prevalence but do not establish independent age-specific biological effects. Third, covariate adjustment for survival outcomes also varied, and HRs reconstructed from survival curves may have introduced measurement error. Fourth, potential dependence arising from shared reference groups was addressed through within-study combination and sensitivity analyses. Although the pooled estimates remained stable, residual correlation cannot be excluded. Fifth, potential dependence arising from overlapping database populations was addressed through sensitivity analyses. Although the pooled estimates remained stable, residual correlation or cohort overlap cannot be excluded. Accordingly, the reported total sample size represents the sum of the included study populations rather than a confirmed number of unique individuals. Sixth, several outcomes were also informed by few studies, limiting subgroup analyses and assessment of publication bias. Seventh, high heterogeneity in OS and CSS remained unexplained, and future studies should prioritize more specific endpoints such as CSS and recurrence-related outcomes. Furthermore, reliance on a single NOS total score may have oversimplified risk-of-bias assessment across the three analytic domains. Although the NOS provides a pragmatic and standardized approach, a composite score and fixed cutoff of 7 cannot ensure unbiased estimates. NOS-based sensitivity and subgroup analyses should therefore be interpreted cautiously, and future meta-analyses should consider domain-specific risk-of-bias tools. In particular, a single NOS total score and a fixed cutoff of 7 may have obscured domain-specific bias and did not adequately capture confounding by indication in treatment comparisons. Finally, manual citation tracking and gray-literature searches were not performed, conference abstracts were excluded, and publication-type exclusions in the Web of Science search may have omitted a small number of eligible studies.

## 5. Conclusions

By integrating molecular, treatment-related, and prognostic evidence, this review clarifies what can—and cannot—currently be concluded about EOGC. Across the included studies, EOGC showed lower observed frequencies of HER2 positivity, MSI/dMMR positivity, and p53 protein overexpression; TP53 mutation frequency was numerically lower but was not statistically significantly reduced. However, because most estimates were unadjusted, these findings describe between-group differences in biomarker prevalence rather than associations independent of confounding. An association between EOGC and E-cadherin loss emerged only in analyses restricted to studies using uniform immunohistochemical criteria, underscoring the need to harmonize assays and scoring methods. Biomarker testing should therefore follow established clinical indications regardless of age, as HER2-positive and MSI/dMMR-positive tumors still occur in subsets of younger patients.

Curative-intent resection was associated with lower mortality; however, confounding by indication, a prediction interval spanning the null, and evidence of very low certainty preclude causal inference. Consequently, these findings do not support an EOGC-specific surgical strategy. Likewise, associations between chemotherapy and survival, together with comparative estimates for OS, DFS, and CSS, were insufficiently robust to inform treatment or surveillance decisions based on age alone.

**Figure 6:**
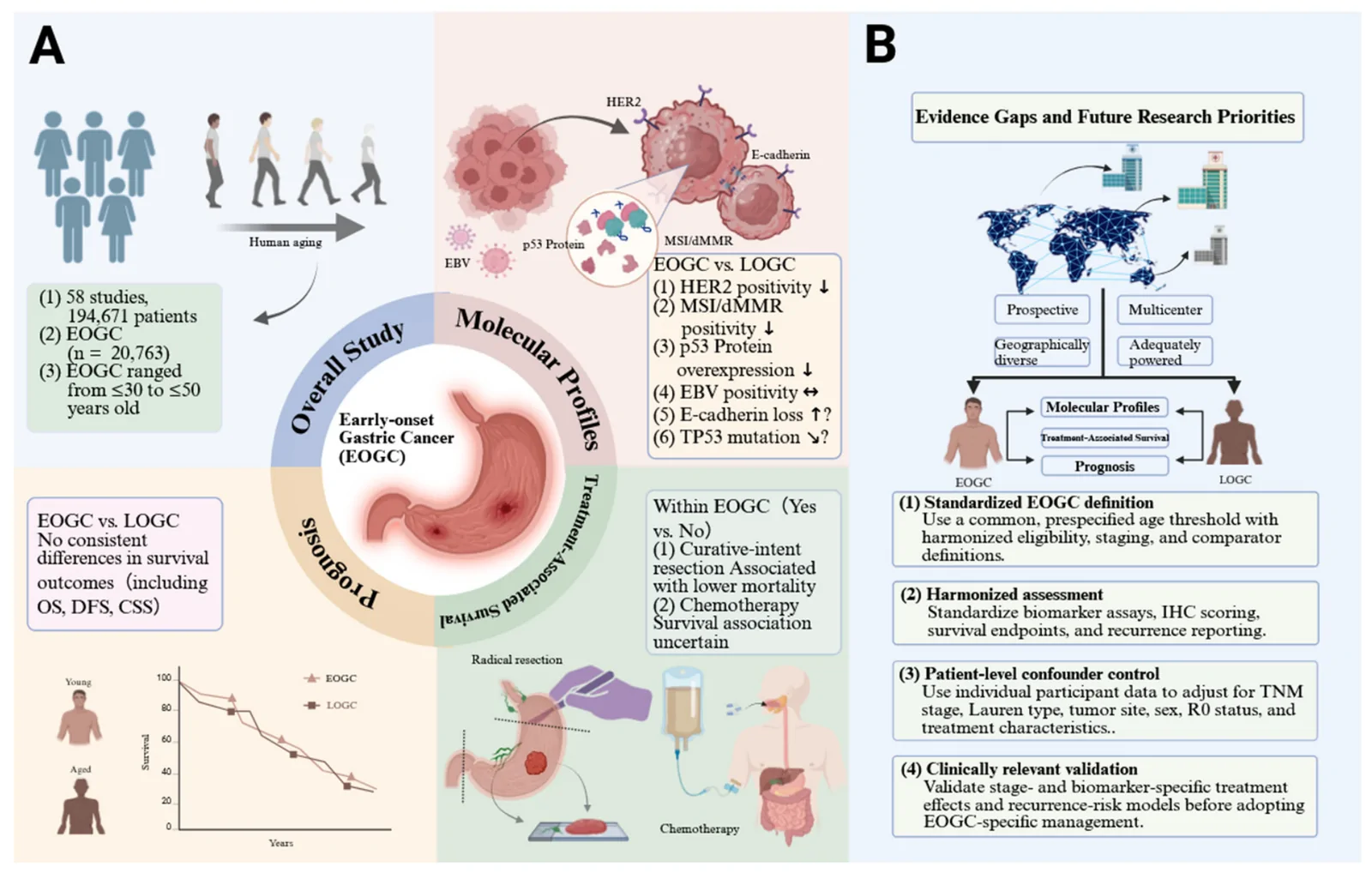
Main findings and future research priorities in early-onset gastric cancer. CSS, cancer-specific survival; DFS, disease-free survival; EOGC, early-onset gastric cancer; LOGC, late-onset gastric cancer; OS, overall survival; HER2, human epidermal growth factor receptor 2; MSI, microsatellite instability; dMMR, deficient mismatch repair; TP53, tumor protein p53; EBV, Epstein–Barr virus; E-cadherin, epithelial cadherin. (**A**) EOGC showed lower HER2, MSI/dMMR, and p53 positivity than LOGC, whereas TP53 mutation, EBV positivity, and E-cadherin loss remained uncertain. Curative-intent resection was associated with lower mortality, but the chemotherapy association was inconclusive. No consistent differences in OS, DFS, or CSS were observed. (**B**) Future studies should standardize EOGC definitions and assessments, control patient-level confounding, and conduct prospective multicenter validation.

The present synthesis distinguishes clinically interpretable findings from hypothesis-generating signals and defines the methodological standards required for further validation. Current evidence does not support separate management based solely on early age at onset. Future prospective multicenter studies should use a common, prespecified age threshold, harmonized eligibility and staging definitions, standardized biomarker and outcome assessments, comparable treatment contexts, and consistent adjustment for major prognostic confounders. The principal findings and future research priorities are summarized in Figure 6.

## Figures and Tables

**Table 1:** Summary characteristics of the included studies.

Characteristic	Category	Value
Included studies	Total	58
Publication year	Range	1996–2025
Data collection period	Range	1965–2024
Total sample size	Patients	194,671
EOGC sample size	Patients	20,763
Sample size per study	Median (IQR; range)	881 (312–3020; 74–33,236)
EOGC sample size per study	Median (IQR; range)	130 (63–270; 13–3641)
Study design	Retrospective cohort	50 (86.2%)
	Retrospective case–control	6 (10.3%)
	Prospective cohort	2 (3.4%)
Data source	Hospital-based	50 (86.2%)
	Database-based	7 (12.1%)
	Mixed hospital/database	1 (1.7%)
Geographic region	Asia	39 (67.2%)
	Americas	11 (19.0%)
	Europe	5 (8.6%)
	Inter-regional/multinational	3 (5.2%)
EOGC age cutoff	≤30 years	3 (5.2%)
	31–40 years	26 (44.8%)
	41–45 years	18 (31.0%)
	46–50 years	11 (19.0%)
Molecular endpoints	HER2	12 studies
	p53 overexpression	8 studies
	TP53 mutation	4 studies
	MSI/dMMR	10 studies
	EBV	5 studies
	E-cadherin	6 studies
Treatment endpoints	Radical/curative resection	9 studies
	Chemotherapy	5 studies
Prognostic endpoints	Overall survival	21 studies
	Disease-free survival	5 studies
	Cancer-specific survival	4 studies
Study quality	NOS score, mean (range)	7.26 (5–9)
	High-quality studies, NOS ≥ 7	49 (84.5%)

EBV, Epstein–Barr virus; EOGC, early-onset gastric cancer; HER2, human epidermal growth factor receptor 2; IQR, interquartile range; MSI/dMMR, microsatellite instability/deficient mismatch repair; NOS, Newcastle–Ottawa Scale; TP53, tumor protein p53 gene. Percentages were calculated using the total number of included studies as the denominator unless otherwise specified. Studies could contribute to more than one molecular, treatment, or prognostic endpoint; therefore, endpoint counts are not mutually exclusive. High-quality studies were defined as those with an NOS score of ≥7. Analytical domains are not mutually exclusive. A total of 25, 14, and 24 studies contributed molecular, treatment, and prognostic endpoints, respectively. The sum of these domain-specific counts (*n* = 63) exceeds the number of unique studies (*n* = 58) because five studies contributed to two domains and were counted in both of the corresponding totals.

**Table 2:** Pooled Effect Estimates, Sensitivity Analyses, and Certainty of Evidence for Key Outcomes.

Outcomes	No. of Studies	Effect Model	OR/HR (95% CI)	I^2^	Sensitivity Analysis Conclusion	GRADE Certainty
HER2	12	Random	OR = 0.66 (0.53–0.84)	19%	Consistent across leave-one-out analysis and after excluding low-quality studies	⊕⊕○○ Low
p53	8	Random	OR = 0.69 (0.60–0.80)	42%	Nonsignificant after excluding low-quality studies (NOS ≤ 6)	⊕⊕○○ Low
TP53	4	REML-KH	OR = 0.60 (0.28–1.29)	25%	Nonsignificant after excluding one study (Dhobi et al., 2013) [23]	⊕⊕○○ Low
MSI/dMMR	10	Random	OR = 0.33 (0.12–0.91)	68%	Non-significant after excluding three individual studies	⊕⊕○○ Low
EBV	5	REML-KH	OR = 1.19 (0.41–3.49)	68%	Consistent across leave-one-out analysis	⊕⊕○○ Low
E-cadherin	6	Random	OR = 2.23 (0.86–5.76)	89%	Estimate dependent on analytical model and single study; heterogeneity resolved under uniform IHC criteria	⊕○○○ Very Low
Radical resection	9	Random	HR = 0.37 (0.22–0.61)	61%	Consistent across leave-one-out analysis and after excluding low-quality studies; 95% PI crossed 1.0	⊕○○○ Very Low
Chemotherapy	5	REML-KH	HR = 0.73 (0.52–1.02)	0%	Nonsignificant after excluding one study (An et al., 2023) [24]	⊕○○○ Very Low
OS	21	Random	HR = 0.88 (0.76–1.02)	90%	Estimate influenced by analytical model and one study; heterogeneity unexplained by meta-regression	⊕○○○ Very Low
DFS	5	REML-KH	HR = 1.24 (0.82–1.88)	57%	Estimate influenced by region and single-study exclusion	⊕○○○ Very Low
CSS	4	REML-KH	HR = 0.87 (0.65–1.15)	92%	Estimate dependent on single study exclusion	⊕○○○ Very Low

OR, odds ratio; HR, hazard ratio; CI, confidence interval; OS, overall survival; DFS, disease-free survival; CSS, cancer-specific survival. ORs were used for molecular endpoints, and HRs were used for treatment and prognostic endpoints.

## Data Availability

The extracted effect estimates, study-level characteristics, and analytic code used for all pooled analyses are available from the corresponding author upon reasonable request. Due to the nature of the included studies (published aggregate data), no individual participant data were used.

## Supplementary Materials

The following supporting information can be downloaded at: https://www.mdpi.com/article/10.3390/jcm15207814/s1, Table S1. Search strategy of four databases. Table S2. Table of basic characteristics of included studies. Table S3. The NOS score of the included studies. Table S4. Results of inter-rater consistency test. Table S5. Results of meta-regression analysis of OS. Table S6. The main treatment mode of patients in 21 studies. Table S7. Sensitivity analysis of pooled effect estimates using restricted maximum likelihood (REML) estimation with Knapp–Hartung adjustment and 95% prediction intervals. Table S8. GRADE Summary of Findings for All Analyzed Outcomes. Table S9. Sensitivity analysis excluding correlated estimates with shared comparators. Table S10. Pooled effect estimates using restricted maximum likelihood (REML) estimation with Knapp–Hartung adjustment. Table S11. PRISMA 2020 Checklist. Supplementary Note: Reasons for exclusion at the full-text review stage. Supplementary Methods: Reconstruction of hazard ratios from published survival curves.
