# Supplementary material for: Molecular Profiles, Treatment-Associated Survival, and Prognosis in Early-Onset Versus Late-Onset Gastric Cancer: A Systematic Review and Meta-Analysis
Source: J Clin Med. 2026 Oct 10;15(20):7814. doi: 10.3390/jcm15207814 (PMC13704902; doi:10.3390/jcm15207814)
Supplement: Supplementary file 1 [file jcm-15-07814-s001.zip › jcm-4521880-supplementary.pdf]

Table S1. Search strategy of four databases.

Databases	Search terms	Number
PubMed	(((((((((("Stomach Neoplasms"[MeSH Terms]) OR (Stomach Neoplasms[Title/Abstract])) OR (Stomach Neoplasm[Title/Abstract])) OR (Gastric Neoplasms[Title/Abstract])) OR (Gastric Neoplasm[Title/Abstract])) OR (Cancer of Stomach[Title/Abstract])) OR (Stomach Cancers[Title/Abstract])) OR (Gastric Cancer[Title/Abstract])) OR (Gastric Cancers[Title/Abstract])) AND ((((Young[Title/Abstract]) OR (Early onset[Title/Abstract])) OR (Early-onset[Title/Abstract])) OR (Young onset[Title/Abstract]))) NOT (Review[Publication Type] OR Meta-analysis[Publication Type] OR Case report[Publication Type] OR Letter[Publication Type] OR Guideline[Publication Type] OR Conference[Publication Type] OR protocol[Publication Type]) AND (1990:2025[pdat])	1,147
Web of Science Core Collection	#1: (((((((TS=("Stomach Neoplasms")) OR TS=(Stomach Neoplasm)) OR TS=(Gastric Neoplasms)) OR TS=(Gastric Neoplasm)) OR TS=(Cancer of Stomach)) OR TS=(Stomach Cancers)) OR TS=(Gastric Cancer)) OR TS=(Gastric Cancers)<br>#2: (((TS=(Young)) OR TS=(Early onset)) OR TS=(Early-onset)) OR TS=(Young onset)<br>#3: ((((((TS=(Review)) OR TS=(Meta analysis)) OR TS=(Letter)) OR TS=(Case report)) OR TS=(Guideline)) OR TS=(Conference)) OR TS=(protocol)<br>#4: #1AND #2<br>#5: (#4) NOT #3<br>#6: #5 AND DOP=(1990-01-01/2025-11-01)	2,441
Embase	#1: 'stomach cancer'/exp<br>#2: 'stomach cancer':ab,ti OR 'stomach neoplasms':ab,ti OR 'stomach neoplasm':ab,ti OR 'gastric neoplasms':ab,ti OR 'gastric neoplasm':ab,ti OR 'cancer of stomach':ab,ti OR 'stomach cancers':ab,ti OR 'gastric cancer':ab,ti OR 'gastric cancers':ab,ti<br>#3: young:ab,ti OR 'early onset':ab,ti OR 'young onset':ab,ti<br>#4: review:it OR 'meta analysis':it OR 'case report':it OR letter:it OR guideline:it OR conference:it OR protocol:it<br>#5: #1 OR #2<br>#6: #3 AND #5	1,445

	#7: #6 NOT #4<br>#8: #7 AND [1990-2025]/py	
Cochrane Library	#1: MeSH descriptor: [Stomach Neoplasms] explode all trees<br>#2: (Stomach Neoplasms):ti,ab,kw OR (Stomach Neoplasm):ti,ab,kw OR (Gastric Neoplasms):ti,ab,kw OR (Gastric Neoplasm):ti,ab,kw OR (Cancer of Stomach):ti,ab,kw (Word variations have been searched)<br>#3: (Stomach Cancers):ti,ab,kw OR (Gastric Cancer):ti,ab,kw OR (Gastric Cancers):ti,ab,kw (Word variations have been searched)<br>#4: (Young):ti,ab,kw OR (Young onset):ti,ab,kw OR (Early onset):ti,ab,kw OR (Early-onset):ti,ab,kw (Word variations have been searched)<br>#5: #1 OR #2 OR #3<br>#6: #4 AND #5<br>#7: #6 with Cochrane Library publication date Between Jan 1990 and Nov 2025	426

Table S2. Table ofbasic characteristics ofincluded studies.

Study	Country	Study design	Data source	Period	Number of cases	Number of EOGC	Early-onset definition (y)	Age categories (y)	Molecular detection methods and judgment criteria	Molecular ratio (Number of occurrences / Total number)	Hazard ratios and 95% confidence intervals ofthe treatment methods	Hazard ratios and 95% confidence intervals ofthe prognosis situation	The Hazard ratios that were ultimately included in the analysis	NOS score
Stryelkina et al., 2025[1]	United States	Retrospective cohort study	Hospital	2002-2022	437	32	< 50	EOGC: <50; LOGC: ≥50	HER2: IHC; if inconclusive, confirm by FISH; 3+ positive; MSI: IHC detectio	HER2: 1/24; MMR deficiency: 2/24	/	/	/	7

									n of MMR protein deficiency					
Lu et al., 2025[2]	China	Retrospective cohort study	Hospital	2013-2018	462	101	≤ 32	EOGC: ≤32; LOGC: >32	/	/	/	OS (EOGC vs LOGC): HR = 0.63, 95% CI: 0.43–0.90; DFS (LOGC vs EOGC): HR = 0.60, 95% CI: 0.41–0.86	OS (EOGC vs LOGC): HR = 0.63, 95% CI: 0.43–0.90; DFS (EOGC vs LOGC): HR = 1.68, 95% CI: 1.16-2.24	8
Liu et al., 2025[3]	China	Retrospective cohort	Hospital	2000-2022	18,877	2,206	≤ 45	EOGC: ≤45; LOGC:	HER2: IHC 3+ or 2+	HER2: 47/1020;	/	/	/	7

		study						>45	and FISH+; dMMR: IHC detection of MMR protein deficiency	dMMR: 4/198				
Liang et al., 2025[4]	China	Retrospective cohort study	Hospital	2009-2019	3,940	572	< 45	EOGC: <45; COGC: 46-70; LOGC: >70	HER2: IHC, IHC 3+ is positive	HER2: 12/331	/	OS (EOGC vs COGC): HR=0.81, 95% CI: 0.66-0.99	OS (EOGC vs COGC): HR=0.81, 95% CI: 0.66-0.99	8
Kamio et al., 2025[5]	Japan	Retrospective cohort study	Database	2019-2024	1,284	143	< 40	EOGC: < 40; LOGC: ≥65	HER2: IHC 3+ is positive; TP53: Detecte	HER2: 19/131; TP53 mutation: 99/143;	/	/	/	7

									d via the CGP panel; MSI: Detected via the CGP panel	MSI-H: 0/143				
Deng et al., 2025[6]	China	Retrospective cohort study	Hospital	2014-2021	887	130	≤ 45	EOGC: ≤45; LOGC: >45	/	/	Chemotherapy (yes vs. no): HR = 0.80, 95% CI: 0.45 – 1.43	/	Chemotherapy (yes vs. no): HR = 0.80, 95% CI: 0.45 – 1.43	8
Zhang et al., 2024[7]	China	Retrospective cohort study	Database	2010-2015	5,580	237	≤ 40	EOGC: ≤40; LOGC: >40	/	/	/	OS (EOGC vs LOGC): HR= 1.303, 95% CI: 1.073-1.582	OS (EOGC vs LOGC): HR= 1.303, 95% CI: 1.073-1.582	8
Han et	China	Retrosp	Databas	/	423	28	< 50	EOGC:	TP53:	TP53	/	/	/	6

al., 2024[8]		ective cohort study	e					<50; LOGC: ≥50	Whole-exome sequencing analysis of somatic mutations; CDH-1: Whole-exome sequencing analysis of somatic mutations	mutations: 10 out of 28; CDH1 mutations: 6 out of28				
Zhong et al., 2023[9]	China	Retrospective cohort study	Hospital	2011-2021	100	100	≤ 30	/	/	/	Radical resection (yes vs. no): HR = 0.130, 95% CI:	/	Radical resection (yes vs. no): HR = 0.130, 95% CI:	7

											0.041–0.411		0.041–0.411	
Yamamoto et al., 2023[10]	Japan	Retrospective cohort study	Hospital	2008-2015	555	20	< 40	EOGC: <40; LOGC: 60-75	/	/	/	OS (EOGC vs LOGC): HR = 1.88, 95% CI: 1.17-3.04	OS (EOGC vs LOGC): HR = 1.88, 95% CI: 1.17-3.04	8
Rompen et al., 2023[11]	Germany	Retrospective cohort study	Hospital	2002-2021	738	129	< 50	EOGC: <50; LOGC: >50	/	/	/	OS (EOGC vs LOGC): HR=0.8, 95% CI: 0.6–1. 1; DFS (EOGC vs LOGC): HR= 1. 1, 95% CI: 0.78–1.4	OS (EOGC vs LOGC): HR=0.8, 95% CI: 0.6–1. 1; DFS (EOGC vs LOGC): HR= 1. 1, 95% CI: 0.78–1.4	8
Liu et	China	Retrosp	Hospital	2003-20	126	126	< 40	/	/	/	Radical	/	Radical	6

al., 2023[12]		ective cohort study		19							resection (No vs. Yes): HR = 1.916, 95% CI: 1.051–3.491		resection (Yes vs. No): HR = 0.52, 95% CI: 0.29–0.95	
Liu et al., 2023[13]	China	Retrospective case-control study	Hospital	2020-2021	2,506	198	< 45	EOGC: <45; LOGC: >45	HER2: IHC 3+ or IHC 2+ and FISH+; dMMR: IHC detection of MMR protein deficiency; EBV: ISH	HER2: 17/198; dMMR: 2/198; EBV: 8/198	/	/	/	7
Chu et al., 2023[14]	China	Retrospective cohort	Hospital	1994-2019	1,959	202	15-39	EOGC: 15-39; LOGC:	/	/	/	OS(EOGC vs LOGC)	OS (EOGC vs	9

		study						>39				HR= 1.586, 95% CI: 1.134-2.219	LOGC) HR= 1.586, 95% CI: 1.134-2.219	
Calderillo-Ruíz et al., 2023[15]	Mexico	Retrospective cohort study	Hospital	2004-2020	2,543	380	≤ 40	EOGC: ≤40; LOGC: ≥41	HER2: IHC 2+ or higher is considered positive	HER2: 8/53	/	OS(EOGC vs LOGC): HR= 1.88, 95% CI: 1.62-2. 19	OS (EOGC vs LOGC): HR= 1.88, 95% CI: 1.62-2. 19	7
An et al., 2023[16]	United States	Retrospective cohort study	Database	2004-2018	3,641	3,641	≤ 50	/	/	/	Chemotherapy (Yes vs. No): HR = 0.640, 95% CI 0.512–0.801	/	Chemotherapy (yes vs. no): HR = 0.640, 95% CI 0.512–0.801	8
An et al., 2022[17]	China	Retrospective cohort	Hospital	2010-2015	686	52	≤ 40	EOGC: ≤40; LOGC:	/	/	Radical resection (yes	/	Radical resection (yes	8

		study						>40			vs. no): HR = 0.041, 95% CI: 0.003 − 0.671		vs. no): HR = 0.041, 95% CI: 0.003 − 0.671	
Machlowska et al., 2021[18]	Poland, the Netherlands, Finland	Retrospective cohort study	Hospital	/	196	107	≤ 45	EOGC: ≤45; LOGC: >45	p53: IHC, positive in ≥30% oftumor cells showing strong nuclear staining; TP53: NGS sequencing	p53: 29/91	/	/	/	6
Huang et al., 2021[19]	China	Retrospective cohort study	Hospital	2009-2019	312	154	≤ 45	EOGC: ≤45; LOGC: >50	HER2: IHC; IHC 2+ or higher is consider	HER2: 12/154	/	/	/	9

									ed positive					
Cheng et al., 2021[20]	China	Retrospective cohort study	Hospital	2013-2017	558	51	≤ 45	EOGC: ≤45; LOGC: >45	/	/	/	OS (EOGC vs LOGC) HR= 1.349, 95% CI: 0.529–3.439; DFS (EOGC vs LOGC) HR= 1.303, 95% CI: 0.520–3.269	OS (EOGC vs LOGC) HR= 1.349, 95% CI: 0.529–3.439; DFS (EOGC vs LOGC) HR= 1.303, 95% CI: 0.520–3.269	9
Zhang et al., 2020[21]	China	Retrospective case-control study	Hospital	2000-2017	14,794	63	10-24	EOGC: 10-24; LOGC: >40	/	/	Radical resection (yes vs. no): HR = 0.54,	/	Radical resection (yes vs. no): HR = 0.54,	7

											95% CI: 0.22–1.30		95% CI: 0.22–1.30	
Moore et al., 2020[22]	Israel, Germany	Retrospective cohort study	Hospital	1997-2016	74	39	< 45	EOGC: <45; LOGC: ≥55	HER2: IHC 3+ or IHC 2+ with positive FISH; MSI: IHC detection of MMR protein loss; EBV: qPCR	HER2: 2/39; MSI-H: 0/33; EBV: 13/39	/	/	/	7
Cheng et al., 2020[23]	China	Retrospective cohort study	Hospital	2000-2015	1,131	270	18-49	EOGC: 18-49; LOGC: 50-59	/	/	/	OS (EOGC vs LOGC): HR= 1.347, 95% CI: 1.018–1.	OS (EOGC vs LOGC): HR= 1.347, 95% CI: 1.018–1.	8

												781; DFS (EOGC vs LOGC): HR= 1.601, 95% CI: 1.079–2.376	781; DFS (EOGC vs LOGC): HR= 1.601, 95% CI: 1.079–2.376	
Yu et al., 2019[24]	South Korea	Retrospective cohort study	Hospital	2004-2013	1,633	341	≤ 45	EOGC: ≤45; LOGC: >45	EBV: IHC	EBV: 10/164	/	/	/	7
Ramos et al., 2019[25]	Brazil	Retrospective cohort study	Hospital	2009-2017	875	84	≤ 45	EOGC: ≤45; LOGC: >45	/	/	/	DFS (LOGC vs EOGC): HR= 1.33, 95% CI: 0.80-2.20	DFS (EOGC vs LOGC): HR=0.75, 95% CI: 0.45-1.25	8
Li et al., 2019[26]	China	Retrospective cohort	Hospital	2012-2017	324	108	≤ 45	EOGC: ≤45; LOGC:	HER2: Unknown;	HER2: 12/108; EBV:	/	/	/	6

		study						>45	EBV: IHC	5/108				
Zhang et al., 2018[27]	China, United States	Retrospective cohort study	Hospital, Database	China: 2000-2012; United States: 2004-2012	China: 1,037; United States: 16,856	China: 244; United States: 1,338	50	EOGC: <50; LOGC: ≥50	/	/	/	OS (LOGC vs EOGC): HR= 1.79, 95% CI: 1.39-2.307; CSS (LOGC vs EOGC): HR= 1.32, 95% CI: 1.20-1.45	OS (EOGC vs LOGC): HR=0.56, 95% CI: 0.44-0.73; CSS (EOGC vs LOGC): HR=0.72, 95% CI: 0.64-0.81	8
Cormedi et al., 2018[28]	Brazil	Retrospective cohort study	Hospital	2011-2013	294	71	≤ 40	EOGC: ≤40; LOGC: 41–65 and ≥66	/	/	Chemotherapy (Yes vs. no): HR= 1. 19, 95%	/	Chemotherapy (Yes vs. no): HR= 1. 19, 95%	7

											CI: 0.42–3.36		CI: 0.42–3.36	
Braga-Neto et al., 2018[29]	Brazil	Prospective cohort study	Hospital	2008-2014	207	41	≤ 45	EOGC: ≤45; LOGC: >45	/	/	/	OS (LOGC vs EOGC): HR= 1.507, 95% CI: 0.910-2.496	OS (EOGC vs LOGC): HR=0.66, 95% CI: 0.40-1.10	7
Song et al., 2017[30]	China	Retrospective cohort study	Hospital	2007-2011	470	112	≤ 40	EOGC: ≤40; LOGC: ≥70	/	/	/	OS (LOGC vs EOGC): HR= 1.818, 95% CI: 1.383 − 2.391	OS (EOGC vs LOGC): HR=0.55, 95% CI: 0.42 −0.72	8
Wang et al., 2016[31]	China	Retrospective cohort study	Hospital	2005-2010	3,930	342	≤ 40	EOGC: ≤40; LOGC: >40	/	/	/	OS (LOGC vs EOGC): HR= 1.7	OS (EOGC vs LOGC): HR=0.5	8

												94, 95% CI: 1.411-2.282	6, 95% CI: 0.44-0.71	
Song et al., 2016[32]	United States	Retrospective cohort study	Database	1988-2010	10,092	1,402	<30, 30-39, 40-49	EOGC: <30, 30–39, 40–49; LOGC: ≥50	/	/	/	CSS (using the 50–59 age group as a reference): <30 years old, HR = 0.93, 95% CI: 0.65–1.34; 30–39 years old, HR = 0.89, 95% CI: 0.75–1.05;	CSS (EOGC vs LOGC): HR= 1.09, 95% CI: 1.00-1.19	7

												40–49 years old, HR = 0.93, 95% CI: (0.83–1.03)		
Liu et al., 2016[33]	China	Retrospective cohort study	Hospital	2008-2014	1,294	198	≤ 40	EOGC: ≤40; LOGC: 55-64	/	/	/	OS (EOGC vs LOGC): HR=0.999, 95% CI: 0.727-1.372	OS (EOGC vs LOGC): HR=0.999, 95% CI: 0.727-1.372	7
Seo et al., 2015[34]	South Korea	Retrospective cohort study	Hospital	2003-2010	1,658	202	≤ 45	EOGC: ≤45; LOGC: >45	HER2: IHC 2+ or higher is considered positive; p53: IHC staining	HER2: 6/202; p53: 44/202; MSI-H: 1/202	/	/	/	8

									of>10% oftumor cells is considered positive; MSI: PCR testing of5 marker loci; ≥2 altered loci is classified as MSI-H					
Gurzu et al., 2015[35]	Romania	Retrospective cohort study	Hospital	2006-2013	191	37	≤ 45	EOGC: ≤45; LOGC: >45	HER2: IHC, using gastric cancer-specific criteria, with "+++"	HER2: 4/37; p53: 15/37; E-cadherin: 10/37	/	/	/	6

									defined as positive; p53: IHC, defined as positive if>10% oftumor cells show membrane, cytoplasmic, or nuclear expression; E-cadherin: IHC, defined as positive if>10%					

									oftumor cells show membrane, cytoplasmic, or nuclear expression					
Schildberg et al., 2014[36]	Germany	Retrospective cohort study	Hospital	1995-2005	80	40	< 50	EOGC: <50; LOGC: ≥50	p53: IHC; E-cadherin: IHC	p53: 13/36; E-cadherin: 39/40	/	/	/	8
Karim et al., 2014[37]	India	Retrospective cohort study	Hospital	/	103	13	< 40	EOGC: <40; LOGC: ≥40	p53: PCR-SSCP and IHC	p53: 2/13	/	/		5
Zheng et al., 2013[38]	China	Retrospective cohort study	Hospital	2004-2006	717	63	≤ 40	EOGC: ≤40; LOGC: >40	/	/	Radical resection (No vs. Yes): HR = 3.177, 95% CI:	/	Radical resection (Yes vs. No): HR = 0.31, 95% CI:	7

											1.462 – 6.904		0.15 – 0.68	
Xu et al., 2013[39]	China	Retrospective cohort study	Hospital	2001-2009	993	99	≤ 40	EOGC: ≤40; LOGC: >40	/	/	Radical resection (No vs. Yes): HR = 2.468, 95% CI: 1.205 – 5.055	/	Radical resection (Yes vs. No): HR = 0.41, 95% CI: 0.20 – 0.83	8
David K al., 2013[40]	United States	Retrospective cohort study	Database	1988-2009	30,296	909	< 40	EOGC: <40; LOGC: ≥40	/	/	/	OS, with those under 40 years of age as the reference group: HR for those aged 40–64 = 0.94 (95%	OS (EOGC vs LOGC): HR = 0.93, 95%CI 0.85-1.01	7

												CI: 0.83–1.06); HR for those aged ≥65 = 1.24 (95% CI: 1.10–1.40)		
Liang et al., 2013[41]	China	Retrospective cohort study	Hospital	2003-2007	920	166	< 50	EOGC: <50; LOGC: 50–69 and ≥70	/	/	/	OS: (Age ≥70 years vs <50 years): HR= 1.487, 95% CI 1.149–1.924: (50–69 years vs <50	OS (EOGC vs LOGC): HR=0.79, 95%CI 0.67-0.94	8

												years): HR= 1. 107, 95% CI 0.881–1.391		
Isobe et al., 2013[42]	Japan	Retrospective cohort study	Hospital	1977-2006	3,818	169	≤ 40	EOGC: ≤40; LOGC: >40	/	/	Radical resection (No vs. Yes) HR=6.322, 95% CI: 2.423–21.255	/	Radical resection (Yes vs. No) HR=0.14, 95% CI: 0.05–0.41	7
Dhobi et al., 2013[43]	India	Prospective cohort study	Hospital	2005-2009	502	50	< 40	EOGC: <40; LOGC: >40	p53: Changes in migration patterns were detected by PCR-SSCP and	p53: 7/30	/	/	/	7

									confirmed by sequencing					
Qiu et al., 2011[44]	China	Retrospective cohort study	Hospital	1996-2006	1,000	294	20-50	EOGC: ≤50; LOGC: >50	/	/	Chemotherapy (no vs. yes): HR = 1.504, 95% CI: 0.408–5.544	/	Chemotherapy (Yes vs. No): HR = 0.66, 95% CI: 0.18–2.45	8
Moelans et al., 2011[45]	Netherlands, Poland, Finland, United States	Retrospective cohort study	Hospital	/	199	108	≤ 45	EOGC: ≤45; LOGC: >45	HER2: IHC; IHC 3+ is considered positive	HER2: 0/108	/	/	/	7
Al-Refaie et al., 2011[46]	United States	Retrospective cohort study	Database	1991-2006	33,236	2,757	< 45	EOGC: <45; LOGC: 45–70 and >70	/	/	/	CSS (compared to those under 45 years	CSS (EOGC vs LOGC): HR=0.85 ,95%	8

												ofage): 45–70 years old, HR = 1.12, 95% CI: 1.07–1. 17; over 70 years old, HR = 1.32, 95% CI: 1.26–1.38	CI 0.82-0.89	
Geramizadeh et al., 2010[47]	Iran	Retrospective case-control study	Hospital	2003-2008	86	43	≤ 50	EOGC: ≤50; LOGC: >50	E-cadherin: IHC	E-cadherin: 31/43	/	/		5
Park et al., 2009[48]	South Korea	Retrospective cohort study	Hospital	2000-2005	3,362	654	≤ 45	EOGC: ≤45; LOGC: >45	/	/	Radical resection (No vs. Yes) HR=2.792, 95% CI:	/	Curative resection (Yes vs. No) HR=0.36, 95% CI:	8

											1.066–7.316		0.14–0.94	
Silva et al., 2008[49]	Brazil	Retrospective cohort study	Hospital	1988-2005	515	62	≤ 40	EOGC: ≤40; LOGC: >40	E-cadherin: IHC	E-cadherin: 26/61	/	OS (LOGC vs EOGC): HR= 1.2, 95% CI: 0.8-1.8	OS (EOGC vs LOGC): HR=0.83, 95% CI: 0.56-1.25	8
Lai et al., 2008[50]	South Korea	Retrospective cohort study	Hospital	1987-2004	6,954	883	≤ 40	EOGC: ≤40; LOGC: >40	/	/	/	OS (40 −65) vs. (under 40): HR = 1.241, 95% CI 1.055 − 1.461; (age > 65) vs. <40: HR = 1.978, 95% CI 1.653 −	OS (EOGC vs LOGC) HR=0.66,95%CI 0.58-0.74	7

												2.366		
Kim et al., 2008[51]	South Korea	Retrospective cohort study	Hospital	1993-2000	1,299	175	≤ 40	EOGC: ≤40; LOGC: >40	/	/	/	OS (EOGC vs LOGC): HR = 0.85, 95% CI: 0.60-1.21	OS (EOGC vs LOGC): HR = 0.85, 95% CI: 0.60-1.21	7
Sasao et al., 2006[52]	Japan	Retrospective case-control study	Hospital	1990-2004	134	68	≤ 40	EOGC: ≤40; LOGC: ≥41	MSI: PCR; if additional bands appear in the tumor DNA upon analysis using BAT26, the result is positive.	MSI: 10/65	/	/	/	6
Milne et	Netherla	Retrosp	Hospital	/	204	113	≤ 45	EOGC:	p53:	p53:	/	/	/	7

al., 2006[53]	nds, Finland	ective case-control study						≤45; LOGC: >45	IHC; positive when >30% of cells show strong nuclear staining; E-cadherin: IHC; abnormal when membrane staining is absent or appears in clumps in the cytoplasm	35/113; E-cadherin: 59/113				
Lim et	South	Retrosp	Hospital	1993-19	4, 123	78	≤ 30	EOGC:	p53:	p53:	/	/	/	7

al., 2003[54]	Korea	ective cohort study		99				≤30; LOGC: >30	IHC, >10% of tumor cells show positive nuclear staining; MSI: PCR and fragment analysis ofthe single-nucleotide marker BAT-26; E-cadherin: IHC, >10% of cancer cells	28/80; E-cadherin abnormality: 53/78; MSI: 1/76; EBV: 9/78				

									show nuclear expression, or <10% of cancer cells show positive membrane staining, which is considered loss of membrane expression; EBV: In situ hybridization					
Kim et al.,	South Korea	Retrospective	Hospital	1988-1998	1,833	137	< 36	EOGC: <36;	/	/	/	OS (EOGC	OS (EOGC	7

2003[55]		cohort study						LOGC: ≥36				vs LOGC): HR= 1.038, 95% CI: 0.845-1.107	vs LOGC): HR= 1.038, 95% CI: 0.845-1.107	
Theuer et al., 1998[56]	United States	Retrospective cohort study	Hospital	1984-1994	3,020	167	≤ 40	EOGC: ≤40; LOGC: >40	/	/	/	OS: Using age ≤40 as the reference group, 41–50: HR = 1.13, 95% CI: 0.83–1.5; 51–60: HR = 1.11, 95% CI: 0.85–1.4; 61–70: HR = 1.25,	OS (EOGC vs LOGC): HR=0.82, 95% CI: 0.72-0.94	7

												95% CI: 0.97–1.6 ; 70 and older: HR = 1.46, 95% CI: 1. 1–1.9		
Jee et al., 1997[57]	South Korea	Retrospective case-control study	Hospital	1990-1996	77	40	< 40	EOGC: <40; LOGC: ≥40	MSI: PCR; PCR analysis of6 microsatellite loci with at least one new band was considered positive	MSI: 9/40	/	/	/	5
Maehara et al.,	Japan	Retrospective	Hospital	1965-1991	530	174	≤ 40	EOGC: ≤40；	p53: IHC,	p53: 13/30	/	/	/	7

1996[58]		cohort study						LOGC: ≥70	IHC, >10% of cancer cell nuclei show positive staining					

EOGC: early-onset gastric cancer; LOGC: late-onset gastric cancer; OS: overall survival; DFS: disease-free survival; HR: hazard ratio; CI: confidence interval; NOS: Newcastle-Ottawa Scale; IHC: immunohistochemistry; FISH: fluorescence in situ hybridization; CGP: comprehensive genomic profiling; ISH: in situ hybridization; qPCR: quantitative polymerase chain reaction; PCR: polymerase chain reaction; SSCP: single-strand conformation polymorphism; NGS: next-generation sequencing. Studies could contribute to more than one analytical domain. Among the 58 included studies, 25 contributed molecular data, 14 contributed treatment- associated survival data, and 24 contributed prognostic data; five studies were classified into two domains.

Table S3. The NOS score ofthe included studies.

Cooperative Study			Selection of Research Subjects				Comparability across groups		Outcome Measurement			
Author (Year)	Country	Research Type	Representativeness ofthe exposed group (1 point)	Selection ofthe non-exposed cohort (1 point)	Methods for measuring exposure factors (1 point)	Did the outcome of interest occur before the intervention? (1 point)	Whether the most important confounding factor was controlled for (1 point)	Whether other confounding factors were controlled for (1 point)	Outcome assessment (1 point)	Is the follow-up period long enough? (1 point)	Adequacy of follow-up of cohorts (loss-to-follow-up rate)? (1 point)	Total score : 9
Stryelkina et al., 2025[1]	United States	Retrospective cohort study	1	1	1	1	0	1	1	1	0	7
Lu et al., 2025[2]	China	Retrospective cohort study	1	1	1	1	0	1	1	1	1	8
Liu et al., 2025[3]	China	Retrospective cohort study	1	1	1	1	0	1	1	1	0	7
Liang et al., 2025[4]	China	Retrospective cohort study	1	1	1	1	0	1	1	1	1	8
Kamio et al.,	Japan	Retrospecti	1	1	1	1	0	1	1	1	0	7

2025[5]		ve cohort study									
Deng et al., 2025[6]	China	Retrospective cohort study	1	1	1	1	1	1	1	1	0	8
Zhang et al., 2024[7]	China	Retrospective cohort study	1	1	1	1	1	1	1	1	0	8
Han et al., 2024[8]	China	Retrospective cohort study	1	1	1	1	0	1	1	0	0	6
Zhong et al., 2023[9]	China	Retrospective cohort study	1	0	1	1	0	1	1	1	1	7
Yamamoto et al., 2023[10]	Japan	Retrospective cohort study	1	1	1	1	1	1	1	1	0	8
Rompen et al., 2023[11]	Germany	Retrospective cohort study	1	1	1	1	1	1	1	1	0	8
Liu et al., 2023[12]	China	Retrospective cohort study	1	0	1	1	1	0	1	1	0	6
Chu et al., 2023[14]	China	Retrospective cohort study	1	1	1	1	1	1	1	1	1	9

Calderillo-Ruíz et al., 2023[15]	Mexico	Retrospective cohort study	1	1	1	1	1	0	1	1	0	7
An et al., 2023[16]	China	Retrospective cohort study	1	1	1	1	1	1	1	1	0	8
An et al., 2022[17]	China	Retrospective cohort study	1	1	1	1	0	1	1	1	1	8
Machlowska et al., 2021[18]	Poland, the Netherlands	Retrospective cohort study	1	1	1	1	0	1	1	0	0	6
Huang et al., 2021[19]	China	Retrospective cohort study	1	1	1	1	1	1	1	1	1	9
Cheng et al., 2021[20]	China	Retrospective cohort study	1	1	1	1	1	1	1	1	1	9
Moore et al., 2020[22]	Israel, Germany	Retrospective cohort study	1	1	1	1	0	1	1	1	0	7
Cheng et al., 2020[23]	China	Retrospective cohort study	1	1	1	1	1	1	1	1	0	8
Yu et al., 2019[24]	South Korea	Retrospective cohort	1	1	1	1	1	0	1	1	0	7

		study										
Ramos et al., 2019[25]	Brazil	Retrospective cohort study	1	1	1	1	1	1	1	1	0	8
Li et al., 2019[26]	China	Retrospective cohort study	1	1	1	1	0	0	1	1	0	6
Zhang et al., 2018[27]	China, United States	Retrospective cohort study	1	1	1	1	1	1	1	1	0	8
Cormedi et al., 2018[28]	Brazil	Retrospective cohort study	1	1	1	1	0	1	1	1	0	7
Braga-Neto et al., 2018[29]	Brazil	Prospective cohort study	1	1	1	1	1	0	1	1	0	7
Song et al., 2017[30]	China	Retrospective cohort study	1	1	1	1	1	1	1	1	0	8
Wang et al., 2016[31]	China	Retrospective cohort study	1	1	1	1	1	0	1	1	1	8
Song et al., 2016[32]	China	Retrospective cohort study	1	1	1	1	1	0	1	1	0	7

Liu et al., 2016[33]	China	Retrospective cohort study	1	1	1	1	1	0	1	1	0	7
Seo et al., 2015[34]	South Korea	Retrospective cohort study	1	1	1	1	1	1	1	1	0	8
Gurzu et al., 2015[35]	Romania	Retrospective cohort study	1	1	1	1	0	1	1	0	0	6
Schildberg et al., 2014[36]	Germany	Retrospective cohort study	1	1	1	1	1	1	1	1	0	8
Karim et al., 2014[37]	India	Retrospective cohort study	1	1	1	1	0	0	1	0	0	5
Zheng et al., 2013[38]	China	Retrospective cohort study	1	1	1	1	0	1	1	1	0	7
Xu et al., 2012[39]	China	Retrospective cohort study	1	1	1	1	0	1	1	1	1	8
David K al., 2013[40]	United States	Retrospective cohort study	1	1	1	1	1	0	1	1	0	7
Liang et al., 2013[41]	China	Retrospective cohort	1	1	1	1	1	1	1	1	0	8

		study										
Isobe et al., 2013[42]	Japan	Retrospective cohort study	1	1	1	1	1	0	1	1	0	7
Dhobi et al., 2013[43]	India	Prospective cohort study	1	1	1	1	0	1	1	1	0	7
Qiu et al., 2011[44]	China	Retrospective cohort study	1	1	1	1	1	1	1	1	0	8
Moelans et al., 2011[45]	Netherlands, Poland, Finland, United States	Retrospective cohort study	1	1	1	1	1	1	1	0	0	7
Al-Refaie et al., 2011[46]	United States	Retrospective cohort study	1	1	1	1	1	1	1	1	0	8
Park et al., 2009[48]	South Korea	Retrospective cohort study	1	1	1	1	1	1	1	1	0	8
Silva et al., 2008[49]	Brazil	Retrospective cohort study	1	1	1	1	1	1	1	1	0	8
Lai et al.,	South	Retrospecti	1	1	1	1	1	0	1	1	0	7

2008[50]	Korea	ve cohort study										
Kim et al., 2008[51]	South Korea	Retrospective cohort study	1	1	1	1	1	0	1	1	0	7
Lim et al., 2003[54]	South Korea	Retrospective cohort study	1	1	1	1	1	0	1	1	0	7
Kim et al., 2003[55]	South Korea	Retrospective cohort study	1	1	1	1	0	1	1	1	0	7
Theuer et al., 1998[56]	United States	Retrospective cohort study	1	1	1	1	0	1	1	1	0	7
Maehara et al., 1996[58]	Japan	Retrospective cohort study	1	1	1	1	1	0	1	1	0	7

**Case-control study**			**Selection of Research Subjects**				**Comparability across groups**		**Measurement of Exposure Factors**			
Author (Year)	Country	Research Type	Determine whether the case is	Representativeness ofthe case (1 point)	Choice of Comparison (1 point)	Determination ofthe Control (1	Whether the most important	Whether other confoundin	Identification of Exposure	Use the same method	Non-response rate (1 point)	Total score : 9

			appropriate (1 point)			point)	confounding factor was controlled for (1 point)	g factors were controlled for (1 point)	Factors (1 point)	to determine exposure factors for the case and control groups (1 point)		
Liu et al., 2023[13]	China	Retrospective case-control study	1	1	1	1	0	0	1	1	1	7
Zhang et al., 2020[21]	China	Retrospective case-control study	1	1	1	1	1	0	1	1	0	7
Geramizadeh et al., 2010[47]	Iran	Retrospective case-control study	1	1	1	1	0	0	1	0	0	5
Sasao et al., 2006[52]	Japan	Retrospective case-control study	1	1	1	1	0	0	1	1	0	6

Milne et al., 2006[53]	Netherlands, Finland	Retrospective case-control study	1	1	1	1	0	1	1	1	0	7
Jee et al., 1997[57]	South Korea	Retrospective case-control study	1	0	1	1	0	0	1	1	0	5

Table S4. Results of inter-rater consistency test.

NOS entry	Kappa coefficient	95% CI	p-value	Agreement rate (%)
Exposure group representativeness (sel1)	1.00[1]	/	/	100
Representativeness of the non-exposed group (sel2)	1.00	/	0.0001	100
Exposure Factor Measurement (sel3)	1.00[1]	/	/	100
Ascertainment of outcome (sel4)	1.00[1]	/	/	100
Control the most important factor (comp1)	0.71	0.43/0.99[2]	0.004	86.7
Control of other confounding factors (comp2)	0.53	0.26/0.93[2]	0.039	80.0
Review of the Ending (out1)	1.00[1]	/	/	100
Follow-up time (out2)	1.00	/	0.0001	100
Quality of follow-up (out3)	1.00	/	0.0001	100
**Average Kappa (9 items)**	**0.91**[3]	/	/	/
**Overall Kappa for all entries**	**0.862**	/	<0.001	/
**NOS Total Score (ICC)**	**0.892**	**0.715/0.962**	<0.001	/

[1] Perfect agreement; Kappa values were included in the average as 1.00.

[2] Confidence intervals were calculated based on a normal approximation (using the variance from the `irr` package).

[3] Average calculated after treating Kappa values for perfect agreement as 1.00 (actual mean ≈ 0.91).

Table S5. Results ofmeta-regression analysis ofOS.

5.1 Results ofthe univariate meta-regression

Covariates	Comparison/Unit	Coefficient (95% CI)	p-value	I2 (%)	R2 (%)	QM Test P-Value
Age thresholds (categories)	45 years old vs. 40 years old or younger	-0.086 (-0.623, 0.452)	0.755	92.3	0	0.903
	Aged 50 vs. aged 40 or younger	-0.080 (-0.499, 0.339)	0.707			
Age threshold (continuous)	For every additional year	-0.002 (-0.036, 0.032)	0.918	92.0	0	—
Region	Others vs. Asia	0.074 (-0.263, 0.412)	0.666	91.2	0	—
Year ofpublication	For every additional year	0.011 (-0.010, 0.031)	0.315	91.4	0	—

5.2 Results ofthe Multifactor Meta-regression

Model	Covariates	Comparison/Unit	Coefficient (95% CI)	p-value	I2 (%)	R2 (%)	QM Test P-Value
Model 1 (Age Group + Region + Sample Size)	Age	45 years old vs. 40 years old or younger	-0.093 (-0.659, 0.474)	0.748	91.1	0	0.982
		Aged 50 vs. aged 40 or younger	-0.088 (-0.541, 0.365)	0.704			
	Region	Others vs. Asia	0.084 (-0.307, 0.474)	0.674			

	Sample size	For every 1 standard deviation increase	-0.033 (-0.215, 0. 150)	0.726			
Model 2 (Continuous Age + Region + Sample Size)	Age	For every additional year	-0.002 (-0.039, 0.034)	0.894	90.7	0	0.972
	Region	Others vs. Asia	0.089 (-0.293, 0.471)	0.649			
	Sample size	For every 1 standard deviation increase	-0.026 (-0.202, 0. 151)	0.774			

OS, overall survival; HR, hazard ratio; CI, confidence interval; SD, standard deviation; QM, omnibus test of moderators; R2, proportion of between-study variance explained by the model. (1) Sample size was not included as a univariate moderator because its coefficient was extremely small (—1.53 × 10-6) with p = 0.901, indicating no clinical significance. (2) An R2 value of 0% indicates that the covariate(s) did not explain any between- study heterogeneity. (3) The QM test P-value for the omnibus test of all moderators in a model is reported for categorical variables (age categories) and multivariate models.

Table S6. The main treatment mode ofpatients in 21 studies.

First author, year	EOGC age cut-off(years)	EOGC sample size (n)	Surgery rate	Chemotherapy rate	Stage III+IV	Median follow-up (months)	HR (95% CI) for OS
Lu et al., 2025[2]	≤ 32	101	100%	/	39.6%	101	HR = 0.63 (0.43-0.90)
Liang et al., 2025[4]	< 45	572	100%	66.6%	48.8%	/	HR = 0.81 (0.66-0.99)
Zhang et al., 2024[7]	≤ 40	237	10.5%	77.2%	100%	/	HR = 0.77 (0.63-0.93)
Yamamoto et al., 2023[10]	< 40	20	35%	85%	100%	/	HR = 1.89 (1.17-3.04)
Rompen et al., 2023[11]	< 50	129	100%	74.0%	56.6%	40.6	HR = 0.82 (0.61-1. 10)
Chu et al., 2023[14]	< 40	200	100%	59.5%	42.0%	/	HR = 1.59 (1.13-2.20)
Calderillo-Ruíz et al., 2023[15]	≤ 40	380	I-III: 100% metastatic: 26%	I-III: 96% metastatic: 72%	79%	/	HR = 1.88 (1.62-2. 19)
Cheng et al., 2021[20]	≤ 45	51	100%	7.8%	29.4%	29.5	HR = 1.35 (0.53-3.44)
Cheng et al., 2020[23]	< 50	270	100%	52.2%	43.3%	47.0	HR = 1.35 (1.02-1.78)
Zhang et al., 2018[27]	< 50	244	100%	/	/	58.5	HR = 0.56 (0.44-0.73)
Braga-Neto et al., 2018[29]	≤ 45	41	58.5%	/	73.2%	/	HR = 0.66 (0.40-1. 10)
Song et al., 2017[30]	≤ 40	112	100%	/	68.8%	/	HR = 0.55 (0.42-0.72)
Wang et al., 2016[31]	≤ 40	342	100%	86. 1%	47.7%	/	HR = 0.56 (0.44-0.71)
Liu et al., 2016[33]	≤ 40	198	100%	/	/	/	HR = 1.00 (0.73-1.37)
David K al., 2013[40]	< 40	909	55.3%	/	56.3%	/	HR = 0.93 (0.85-1.01)
Liang et al., 2013[41]	< 50	166	100%	42.8%	61.4%	/	HR = 0.79 (0.67-0.94)
Silva et al., 2008[49]	≤ 40	62	/	/	62.5%	/	HR = 0.83 (0.56-1.25)
Lai et al., 2008[50]	≤ 40	883	100%	/	34.4%	/	HR = 0.66 (0.58-0.74)
Kim et al., 2008[51]	≤ 40	175	/	/	43.4%	/	HR = 0.85 (0.60-1.21)
Kim et al., 2003[55]	< 36	137	73.7%	/	54.8%	/	HR = 0.97 (0.84-1. 11)

Theuer et al., 1998[56]	≤ 40	167	49%	50%	45%	/	HR = 0.82 (0.72-0.94)

EOGC: early-onset gastric cancer; HR: hazard ratio; CI: confidence interval; OS: overall survival.

Table S7. Sensitivity analysis ofpooled effect estimates using restricted maximum likelihood (REML) estimation with Knapp −Hartung adjustment and 95% prediction intervals.

Outcome	Studies	Pooled HR (95% CI)	95% PI	τ^2	I^2 (%)
Radical resection	9	0.368 (0.189–0.716)	0.076–1.772	0.381	64.3
Chemotherapy	5	0.731 (0.524–1.018)	0.448–1.191	0.017	12.7
CSS	4	0.866 (0.654–1. 147)	0.473–1.586	0.028	92.9
DFS	5	1.237 (0.815–1.876)	0.539–2.839	0.067	59.1
OS	21	0.881 (0.745–1.043)	0.429–1.811	0.113	91.6
E-cadherin (all 6 studies)	6	3.325 (0.201–54.928)	0.004–2626.422	5.546	97.6
E-cadherin (excluding Schildberg)	5	1.314 (0.543–3. 183)	0.182–9.505	0.406	78.9

HR, hazard ratio; OR, odds ratio; CI, confidence interval; PI, prediction interval. τ^2 estimated by REML, CIs adjusted using Knapp–Hartung. E-cadherin results are presented as ORs; the analysis excluding Schildberg et al. (2014) was performed as a sensitivity analysis due to its extreme OR (1521) and wide CI.

Table S8. GRADE Summary ofFindings for All Analyzed Outcomes.

Outcome	No. of Studies	Study Limitations[a]	Inconsistency[b]	Indirectness	Imprecision[c]	Publication Bias	Upgrading Factors	GRADE Certainty
Molecular Markers								
HER2 Positivity	12	Serious[1] (-1)	No serious	No serious	No serious	Undetected	None	⊕⊕○○ Low
p53 Overexpression	8	Serious[2] (-1)	No serious	No serious	No serious	Not assessable[12]	None	⊕⊕○○ Low
TP53 Mutation	4	Serious[3] (-1)	No serious	No serious	No serious	Not assessable[12]	None	⊕⊕○○ Low
MSI/dMMR	10	Serious[4] (-1)	No serious	No serious	No serious	Not assessable[12]	None	⊕⊕○○ Low
EBV Positivity	5	No serious	No serious	No serious	Serious[5] (-1)	Not assessable[12]	None	⊕⊕○○ Low
E- cadherin Loss (All)	6	No serious	Very serious[6] (-2)	No serious	Serious (-1)	Not assessable[12]	None	⊕○○○ Very Low
Treatment Outcomes (within EOGC)								
Radical Resection (OS)	9	Very serious[7] (-2)	Serious (-1)	No serious	No serious	Not assessable[12]	None	⊕○○○ Very Low
Chemotherapy (OS)	5	Serious[8] (-1)	No serious	No serious	No serious	Not assessable[12]	None	⊕○○○ Very Low
Prognostic Outcomes (EOGC vs LOGC)								
Overall Survival (OS)	21	No serious	Very serious[9] (-2)	No serious	No serious	Undetected	None	⊕○○○ Very Low
Disease-Free Survival (DFS)	5	Serious[10] (-1)	Serious[10](-1)	No serious	Serious[10] (-1)	Not assessable[12]	None	⊕○○○ Very Low

Cancer-Specific Survival (CSS)	4	No serious	Very serious[11] (-2)	No serious	No serious	Not assessable[12]	None	⊕○○○ Very Low

[a] Based on Newcastle-Ottawa Scale scores (mean 7.26) and results of sensitivity analyses (leave-one-out and low-quality study exclusion).

[b] Based on I^2 statistics ($I^2 > 50\%$ considered serious; $I^2 > 80\%$ considered very serious) and subgroup interaction P-values.

[c] Based on width of 95% CIs and whether they cross the threshold for appreciable benefit (HR < 0.8 or OR < 0.5) or harm (HR > 1.25 or OR > 2.0).

[1] The majority of included studies reported unadjusted comparisons; the observed difference could be partially confounded by the enrichment of diffuse-type histology (which inherently exhibits lower HER2 positivity) in EOGC, rather than reflecting a purely age-specific biological effect.

[2] Sensitivity analysis showed that the association lost significance after excluding low-quality studies (NOS ≤6).

[3] The pooled estimate was non-significant after excluding Dhobi et al. (2013) in leave-one-out analysis.

[4] The association was sensitive to exclusion of three individual studies (Moore et al., 2020; Liu et al., 2023; Seo et al., 2015).

[5] 95% CI (0.58–2.45) crosses both 0.67 and 1.5; only 5 studies available.

[6] I^2=89%; the estimate was highly dependent on statistical model and a single study; however, heterogeneity resolved (I^2= 13%) when analysis was restricted to uniform IHC criteria (<10%).

[7] Downgraded by two levels for very serious risk of bias. All included studies were observational and subject to severe confounding by indication. The comparator groups were clinically heterogeneous (ranging from non-curative surgery to no surgery), and adjustment variables differed substantially across studies. The 95% prediction interval (0.08–1.77) further indicates that the true association could be in the opposite direction in some future settings, failing the criterion for a large effect upgrade.

[8] The pooled estimate was highly sensitive to exclusion of An et al. (2023), a study restricted to metastatic patients.

[9] I^2=90%; the null effect was fragile, becoming significant when the analytical model was changed or one study was excluded; meta-regression did not explain heterogeneity.

[10] Downgraded for inconsistency: Moderate to substantial statistical heterogeneity was detected (I^2 = 57%), and the pooled estimate was significantly influenced by regional differences (P for interaction = 0.008). Although point estimates were directionally consistent, the magnitude of effect and underlying clinical settings (e.g., follow-up protocols, adjuvant treatment intensity) differed markedly between Asian and non-Asian cohorts, justifying a downgrade. 95% CI (0.92–1.67) crosses both 0.8 and 1.25; the estimate is compatible with appreciable benefit and harm. The three Chinese studies were single-center retrospective cohorts, susceptible to selection bias; leave-one-out analyses yielded inconsistent results.

[11] I^2=92%; the estimate was dependent on inclusion/exclusion of a single study (Song et al., 2016).

[12] Not assessable due to too few studies to evaluate publication bias.

Table S9. Sensitivity analysis excluding correlated estimates with shared comparators.

Outcome	Studies with shared comparators excluded	Studies remaining (n)	Pooled estimate before exclusion (95% CI)	Pooled estimate after exclusion (95% CI)	I^2 before / after
Chemotherapy	1	4	HR 0.69 (0.57–0.84)	HR 0.67 (0.55–0.83)	0% / 0%
OS	4	17	HR 0.88 (0.76–1.02)	HR 0.91 (0.74–1. 12)	90% / 91%
CSS	2	2	HR 0.87 (0.74–1.02)	HR 0.75 (0.66–0.85)	92% / 18%

HR, hazard ratio; CI, confidence interval; OS, overall survival; CSS, cancer- specific survival; I^2, heterogeneity statistic.

Table S10. Pooled effect estimates using restricted maximum likelihood (REML) estimation with Knapp-Hartung adjustment.

	Studies	OR/HR	CI_lower	CI_upper	PI_lower	PI_upper	Tau2
HER2	12	0.74	0.541	1.013	0.351	1.559	0.0942
p53	8	0.739	0.588	0.929	0.466	1.172	0.0287
TP53	4	0.602	0.281	1.29	0.184	1.969	0.0813
MSI	10	0.354	0.108	1.157	0.016	7.766	1.5902
EBV	5	1.194	0.408	3.494	0.133	10.715	0.4751
E-cadherin	6	3.325	0.201	54.928	0.004	2626.422	5.5463
Radical resection	9	0.368	0.189	0.716	0.076	1.772	0.3813
Chemotherapy	5	0.731	0.524	1.018	0.448	1.191	0.0167
CSS	4	0.866	0.654	1.147	0.473	1.586	0.0284
DFS	5	1.237	0.815	1.876	0.539	2.839	0.0671
OS	21	0.881	0.745	1.043	0.429	1.811	0.1126

OR, odds ratio; HR, hazard ratio; CI, confidence interval; PI, prediction interval; Tau^2, estimated between-study variance. REML with Knapp–Hartung adjustment was used for all analyses.

Table S11. PRISMA 2020 Checklist.			
**Section and Topic**	**Item #**	**Checklist item**	**Location where item is reported**
**TITLE**			
Title	1	Identify the report as a systematic review.	Title page
**ABSTRACT**			
Abstract	2	See the PRISMA 2020 for Abstracts checklist.	Abstract (page 1)
**INTRODUCTION**			
Rationale	3	Describe the rationale for the review in the context of existing knowledge.	Introduction (page 2)
Objectives	4	Provide an explicit statement of the objective(s) or question(s) the review addresses.	Introduction (page 2)
**METHODS**			
Eligibility criteria	5	Specify the inclusion and exclusion criteria for the review and how studies were grouped for the syntheses.	Methods - Inclusion and Exclusion Criteria (pages 3-4)
Information sources	6	Specify all databases, registers, websites, organisations, reference lists and other sources searched or consulted to identify studies. Specify the date when each source was last searched or consulted.	Methods - Search Strategy (page 3); Table S1
Search strategy	7	Present the full search strategies for all databases, registers and websites, including any filters and limits used.	Methods - Search Strategy (page 3); Table S1
Selection process	8	Specify the methods used to decide whether a study met the inclusion criteria of the review, including how many reviewers screened each	Methods - Data

Table S11. PRISMA 2020 Checklist.			
**Section and Topic**	**Item #**	**Checklist item**	**Location where item is reported**
		record and each report retrieved, whether they worked independently, and if applicable, details of automation tools used in the process.	Extraction and Quality Assessment (page 4)
Data collection process	9	Specify the methods used to collect data from reports, including how many reviewers collected data from each report, whether they worked independently, any processes for obtaining or confirming data from study investigators, and if applicable, details of automation tools used in the process.	Methods - Data Extraction and Quality Assessment (page 4)
Data items	10a	List and define all outcomes for which data were sought. Specify whether all results that were compatible with each outcome domain in each study were sought (e.g. for all measures, time points, analyses), and if not, the methods used to decide which results to collect.	Methods - Data Extraction (page 4)
	10b	List and define all other variables for which data were sought (e.g. participant and intervention characteristics, funding sources). Describe any assumptions made about any missing or unclear information.	Methods - Data Extraction (page 4)
Study risk of bias assessment	11	Specify the methods used to assess risk of bias in the included studies, including details of the tool(s) used, how many reviewers assessed each study and whether they worked independently, and if applicable, details of automation tools used in the process.	Methods - Quality Assessment (NOS) (page 4); Tables S3-S4
Effect measures	12	Specify for each outcome the effect measure(s) (e.g. risk ratio, mean difference) used in the synthesis or presentation of results.	Methods - Data Analysis (pages 4-5)
Synthesis methods	13a	Describe the processes used to decide which studies were eligible for each synthesis (e.g. tabulating the study intervention characteristics and comparing against the planned groups for each synthesis (item #5)).	Methods - Data Analysis (pages 4-5)

Table S11. PRISMA 2020 Checklist.

Section and Topic	Item #	Checklist item	Location where item is reported
	13b	Describe any methods required to prepare the data for presentation or synthesis, such as handling of missing summary statistics, or data conversions.	Methods - Data Analysis (pages 4-5)
	13c	Describe any methods used to tabulate or visually display results of individual studies and syntheses.	Methods - Data Analysis (pages 4-5); Results - Table 1, Table 2, Figures 1-4
	13d	Describe any methods used to synthesize results and provide a rationale for the choice(s). If meta-analysis was performed, describe the model(s), method(s) to identify the presence and extent of statistical heterogeneity, and software package(s) used.	Methods - Data Analysis (pages 4-5)
	13e	Describe any methods used to explore possible causes of heterogeneity among study results (e.g. subgroup analysis, meta-regression).	Methods - Data Analysis (page 5)
	13f	Describe any sensitivity analyses conducted to assess robustness of the synthesized results.	Methods - Data Analysis (page 5)
Reporting bias assessment	14	Describe any methods used to assess risk of bias due to missing results in a synthesis (arising from reporting biases).	Methods - Data Analysis (page 5); Results - Publication Bias (page 11); Figure S1

Table S11. PRISMA 2020 Checklist.

Section and Topic	Item #	Checklist item	Location where item is reported
Certainty assessment	15	Describe any methods used to assess certainty (or confidence) in the body of evidence for an outcome.	Discussion - Limitations (pages 14-15)
**RESULTS**			
Study selection	16a	Describe the results of the search and selection process, from the number of records identified in the search to the number of studies included in the review, ideally using a flow diagram.	Results - Study Selection (pages 5-6); Figure 1
	16b	Cite studies that might appear to meet the inclusion criteria, but which were excluded, and explain why they were excluded.	Results - Study Selection (page 5)
Study characteristics	17	Cite each included study and present its characteristics.	Results - Table 1; Table S2
Risk of bias in studies	18	Present assessments of risk of bias for each included study.	Results - NOS scores (pages 6-7); Tables S3-S4
Results of individual studies	19	For all outcomes, present, for each study: (a) summary statistics for each group (where appropriate) and (b) an effect estimate and its precision (e.g. confidence/credible interval), ideally using structured tables or plots.	Results - Sections 3.2, 3.3, 3.4; Figures 2-4
Results of syntheses	20a	For each synthesis, briefly summarise the characteristics and risk of bias among contributing studies.	Results - Table 2
	20b	Present results of all statistical syntheses conducted. If meta-analysis was done, present for each the summary estimate and its precision (e.g. confidence/credible interval) and measures of statistical heterogeneity. If comparing groups, describe the direction of the effect.	Results - Table 2; Figures 2-4

Table S11. PRISMA 2020 Checklist.			
**Section and Topic**	**Item #**	**Checklist item**	**Location where item is reported**
	20c	Present results of all investigations of possible causes of heterogeneity among study results.	Results - Subgroup analyses (Sections 3.2-3.4)
	20d	Present results of all sensitivity analyses conducted to assess the robustness of the synthesized results.	Results - Sensitivity analyses (Sections 3.2-3.4)
Reporting biases	21	Present assessments of risk of bias due to missing results (arising from reporting biases) for each synthesis assessed.	Results - Publication Bias (page 11); Figure S1
Certainty of evidence	22	Present assessments of certainty (or confidence) in the body of evidence for each outcome assessed.	Discussion - Limitations (pages 14-15)
**DISCUSSION**			
Discussion	23a	Provide a general interpretation of the results in the context of other evidence.	Discussion - Core Findings (pages 12-13)
	23b	Discuss any limitations of the evidence included in the review.	Discussion - Limitations (pages 14-15)
	23c	Discuss any limitations of the review processes used.	Discussion -

Table S11. PRISMA 2020 Checklist.			
**Section and Topic**	**Item #**	**Checklist item**	**Location where item is reported**
			Limitations (pages 14-15)
	23d	Discuss implications of the results for practice, policy, and future research.	Discussion - Clinical Implications (pages 13-14)
**OTHER INFORMATION**			
Registration and protocol	24a	Provide registration information for the review, including register name and registration number, or state that the review was not registered.	Methods - PROSPERO registration CRD420251207966 (page 3)
	24b	Indicate where the review protocol can be accessed, or state that a protocol was not prepared.	Methods - PROSPERO registration (page 3)
	24c	Describe and explain any amendments to information provided at registration or in the protocol.	Not applicable (no amendments)
Support	25	Describe sources of financial or non-financial support for the review, and the role of the funders or sponsors in the review.	Funding (page 16)
Competing interests	26	Declare any competing interests of review authors.	Conflicts of Interest (page 16)
Availability of	27	Report which of the following are publicly available and where they can be found: template data collection forms; data extracted from	Data Availability

Table S11. PRISMA 2020 Checklist.			
**Section and Topic**	**Item #**	**Checklist item**	**Location where item is reported**
data, code and other materials		included studies; data used for all analyses; analytic code; any other materials used in the review.	Statement (page 16) and Supplementary Materials (page 16)

From: Page MJ, McKenzie JE, Bossuyt PM, Boutron I, Hoffmann TC, Mulrow CD, et al. The PRISMA 2020 statement: an updated guideline for reporting systematic reviews. BMJ 2021;372:n71. doi: 10.1136/bmj.n71. This work is licensed under CC BY 4.0. To view a copy of this license, visit https://creativecommons.org/licenses/by/4.0/

Supplementary Note: Reasons for exclusion at the full- text review stage

Of the 1,299 full- text articles assessed, 1,241 were excluded for the reasons listed in Figure 1. The broad category “other research directions” comprised 636 records and was further classified into five specific subcategories: (1) studies focused on cancers other than gastric or gastroesophageal junction adenocarcinoma; (2) treatment or pharmaceutical studies without an early- onset screening or risk- stratification component; (3) investigations of genetic syndromes or rare germline mutations that lacked population- level screening aims; (4) purely basic or mechanistic studies with no human clinical data; and (5) epidemiological or prognostic studies that did not specifically define or analyze an early- onset age group. The remaining 605 records were excluded because of ineligible publication types (n = 307), duplicate populations (n = 82), missing extractable effect estimates (n = 94), and other reasons (n = 122). A study- level list of excluded full- text articles with reasons can be provided on request.

Supplementary Methods: Reconstruction of hazard ratios from published survival curves

For one included study (Deng et al., 2025), the hazard ratio (HR) was not directly reported and was therefore reconstructed from the published Kaplan–Meier curve using the method described by Tierney et al. (2007). Briefly, the curve was digitized using Engauge Digitizer version 12.1, and time- to- event data were extracted over a series of defined time intervals. The numbers of events and patients at risk within each interval were estimated, and interval- specific log HRs and their variances were calculated. These were subsequently pooled across intervals using a fixed- effect model to obtain a single study- level log HR and its standard error. Censoring was accounted for throughout the estimation procedure. All HR reconstructions were conducted independently by two reviewers, and discrepancies were resolved by re- extraction and discussion.

List of included studies

1. Stryelkina M, Nguyen M, Reddy P, Doan AT, Min M, Go L, Wong L. Early onset gastric cancer: clinical and molecular insights into a rising epidemic among younger adults. *Proceedings (Baylor University Medical Center)*. 2025;38(4):388-396. doi:10. 1080/08998280.2025.2488592

2. Lu J, Lv C, Tong L, Chen J, Wu J, Liu F. Analysis ofage cut-offand prognosis ofearly-onset gastric cancer in young patients. *Zhonghua wei chang wai ke za zhi [Chinesejournal ofgastrointestinal surgery]*. 2025;28(4):400-407. doi:10.3760/cma.j.cn441530-20241023-00349

3. Liu Y, Zhang X, Gan L, Chen Z, Wang X, Zhang J, Chen J, Tan C, Sheng W, Xu M. Trends, clinicopathological features, surgical treatment patterns and prognoses of early-onset versus late-onset gastric cancer: A retrospective cohort study. *Journal ofadvanced research.* Sep 2025;75:697-705. doi:10. 1016/j.jare.2024. 11.028

4. Liang Y, Liu K, Kudriashov V, Sun L, Zhang W, Chen X, Zhao L, Song X, Yang K, Hu J. Comparison among Early-Onset, Late-Onset, and Conventional-Onset Adenocarcinoma of Stomach and Esophagogastric Junction: a Retrospective Study. *Worldjournal ofsurgical oncology*. Jun 7 2025;23(1):222. doi:10. 1186/s12957-025-03867-2

5. Kamio T, Kono Y, Hirosuna K, Ozato T, Yamamoto H, Hirasawa A, Ennishi D, Tomida S, Toyooka S, Otsuka M. Genomic Differences and Distinct TP53 Mutation Site-Linked Chemosensitivity in Early- and Late-Onset Gastric Cancer. *Cancer medicine.* Apr 2025;14(8):e70793. doi:10. 1002/cam4.70793

6. Deng Z, Guo J, Xiong Z, Zhong B, Huang D, Xu H, Chen S, Lian L. Age-Stratified Clinicopathological Features and Efficacy ofAdjuvant Chemotherapy in Resectable Gastric Cancer: An East-West Population-Based Study. *Current oncology (Toronto, Ont)*. Aug 26 2025;32(9)doi:10 .3390/curroncol32090480

7. Zhang H, Cheng X, Guo W, Zheng C, Zhang Y, Jing X, Qiao H. Metastasis patterns and prognosis in young gastric cancer patients: A propensity score-matched SEER database analysis. *PloS one*. 2024;19(4):e0301834. doi:10. 1371/journal.pone.0301834

8. Han X, Jia X, Sheng C, Li M, Han J, Duan F, Wang K. A comparison analysis ofthe somatic mutations in early-onset gastric cancer and traditional gastric cancer. *Clinics and research in hepatology and gastroenterology.* Feb 2024;48(2):102287. doi:10. 1016/j.clinre.2024. 102287

9. Zhong N, Yu Y, Chen J, Shao Y, Peng Z, Li J. Clinicopathological characteristics, survival outcome and prognostic factors ofvery young gastric cancer. *Clinical and experimental medicine*. Jun 2023;23(2):437-445. doi:10. 1007/s10238-022-00822-3

10. Yamamoto R, Honda M, Kawamura H, Kobayashi H, Takiguchi K, Muto A, Yamazaki S, Teranishi Y, Shiraso S, Kono K, Hori S, Kamiga T, Iwao T, Yamashita N. Clinical Features and Survival ofYoung Adults with Stage IV Gastric Cancer: a Japanese Population-Based Study. *Journal of gastrointestinal cancer.* Mar 2023;54(1):56-61. doi:10. 1007/s12029-021-00797-6

11. Rompen IF, Nienhüser H, Crnovrsanin N, Musa J, Haag GM, Longerich T, Fiedler T, Müller-Stich BP, Sisic L, Billeter AT. Clinical Characteristics and Oncological Outcomes of Surgically Treated Early-Onset Gastric Adenocarcinoma - a Retrospective Cohort Study. *Journal ofCancer*. 2023;14(9):1470-1478. doi:10.7150/jca.82876

12. Liu L, Lin J, Zhao J, Yan P. Analysis ofclinicopathologic characteristics and prognosis of gastric cancer in patients <40 years. *Medicine.* Aug 25 2023;102(34):e34635. doi:10. 1097/md.0000000000034635

13. Liu J, Ye B, Fu M, Zhang Q, Chen H, Sun J, Cai T, Wang Z, He H, Zhao J, Li H, Wang X, Sun Y.

Clinicopathological and molecular diagnostic features of early-onset gastric cancer: a study based on data from a single-center dedicated gastric cancer database. *Zhonghua wei chang wai ke za zhi [Chinesejournal ofgastrointestinal surgery]* . 2023;26(10):963-967 .

doi:10 .3760/cma.j.cn441530-20230603-00190

14 . Chu H, Chen X, Liu X, Deng C, Bi B, He Y, Huo M, Zhang C. Clinicopathological characteristics and prognosis of adolescents and young adults with gastric cancer after gastrectomy: a propensity score matching analysis. *Frontiers in oncology.* 2023;13:1204400 . doi:10 .3389/fonc.2023. 1204400

15 . Calderillo-Ruíz G, Díaz-Romero MC, Carbajal-López B, Herrera-Martínez M, Ruiz-García E, Leon-Takahashi AM, López-Basave HN, Meneses-García A, Herrera-Gomez Á . Latin American young patients with gastric adenocarcinoma: worst prognosis and outcomes. *Journal ofgastrointestinal oncology.* Oct 31 2023;14(5):2018-2027 . doi:10 .21037/jgo-23-259

16. An H, Wang PY, Liu YC. Palliative Gastrectomy Improves the Survival ofPatients with Metastatic Early-Onset Gastric Cancer: A Retrospective Cohort Study. *Current oncology (Toronto, Ont).* Aug 27 2023;30(9):7874-7890 . doi:10 .3390/curroncol30090572

17. An J, Ma X, Zhang C, Zhou W, Wang C, Miao W, Cai B, Yu P, Qiao W, Xu Z. Comparison of incidence and prognosis between young and old gastric cancer patient in North-Western China: A retrospective cohort study. *Medicine.* Oct 21 2022;101(42):e31255 .

doi:10. 1097/md.0000000000031255

18 . Machlowska J, Kapusta P, Szlendak M, Bogdali A, Morsink F, Wołkow P, Maciejewski R, Offerhaus GJA, Sitarz R. Status of CHEK2 and p53 in patients with early-onset and conventional gastric cancer. *Oncology letters.* May 2021;21(5):348 . doi:10 .3892/ol.2021. 12609

19. Huang Q, Zheng X, Jiao Y, Lei Y, Li X, Bi F, Guo F, Wang G, Liu M. A Distinct Clinicopathological Feature and Prognosis ofYoung Gastric Cancer Patients Aged ≤ 45 Years Old. *Frontiers in oncology.* 2021;11:674224 . doi:10 .3389/fonc.2021.674224

20 . Cheng YX, Tao W, Liu XY, Yuan C, Zhang B, Zhang W, Peng D. The outcome ofyoung vs. old gastric cancer patients following gastrectomy: a propensity score matching analysis. *BMC surgery.* Nov 19 2021;21(1):399. doi:10. 1186/s12893-021-01401-1

21 . Zhang W, Liang W, Cai A, Wang P, Xi H, Liu G, Wei B. Clinicopathological characteristics of gastric cancer in adolescents aged 10-24 years: 17-year experience of 17 years in a single institute. *Zhonghua wei chang wai ke za zhi [Chinesejournal ofgastrointestinal surgery]* . 2020;23(10):963-968 . doi:10 .3760/cma.j.cn.441530-20190918-00351

22 . Moore A, Hikri E, Goshen-Lago T, Barkan T, Morgenstern S, Brook E, Maderer A, Roth W, Gordon N, Kashtan H, Brenner B, Moehler M, Aharon IB. Young-onset gastric cancer and Epstein-Barr Virus (EBV) - a major player in the pathogenesis? *BMC cancer.* Jan 14 2020;20(1):34 .

doi:10. 1186/s12885-020-6517-0

23. Cheng L, Chen S, Wu W, Kuo ZC, Wei Z, Meng S, Chen C, Zhang C, He Y. Gastric cancer in young patients: a separate entity with aggressive features and poor prognosis. *Journal ofcancer research and clinical oncology.* Nov 2020;146(11):2937-2947 . doi:10. 1007/s00432-020-03268-w

24. Yu JI, Lim DH, Lee J, Kang WK, Park SH, Park JO, Lim HY, Kim ST, Kim S, Sohn TS, Lee JH, An JY, Choi MG, Bae JM, Kim HS, Ahn S. Clinical Outcomes and the Role ofAdjuvant Concurrent Chemoradiation Therapy in D2-resected LN-positive Young Patients (≤45 Years) With Gastric Cancer. *Anticancer research.* Oct 2019;39(10):5811-5820. doi:10 .21873/anticanres. 13785

25. Ramos M, Pereira MA, Sagae VMT, Mester M, Morrell ALG, Dias AR, Zilberstein B, Ribeiro Junior U, Cecconello I. Gastric cancer in young adults: a worse prognosis group? *Revista do Colegio*

Brasileiro de Cirurgioes. 2019;46(4):e20192256. Câncer gástrico em adultosjovens: um grupo de pior prognóstico? doi:10. 1590/0100-6991e-20192256

26. Li C, Wang L, Lan Q, Ma X, Guo D, Xu L, Geng J, Liu X. Clinicopathological features ofgastric cancer in 108 young patients. *Xi 'anJiao TongDaXueXue Bao (YiXue Ban)*. 2019;40(6):896-900. doi:10 .7652/jdyxb201906015

27. Zhang J, Gan L, Xu MD, Huang M, Zhang X, Gong Y, Wang X, Yu G, Guo W. The prognostic value ofage in non-metastatic gastric cancer after gastrectomy: a retrospective study in the U.S. and China. *Journal ofCancer*. 2018;9(7):1188-1199. doi:10 .7150/jca.22085

28. Cormedi MCV, Katayama MLH, Guindalini RSC, Faraj SF, Folgueira M. Survival and prognosis ofyoung adults with gastric cancer. *Clinics (Sao Paulo, Brazil)*. Sep 21 2018;73(suppl 1):e651s. doi:10 .6061/clinics/2018/e651s

29. Braga-Neto MB, Carneiro JG, de Castro Barbosa AM, Silva IS, Maia DC, Maciel FS, de Alcântara RJA, Vasconscelos PRL, Braga L. Clinical characteristics ofdistal gastric cancer in young adults from Northeastern Brazil. *BMC cancer*. Feb 5 2018;18(1):131. doi:10. 1186/s12885-018-3995-4

30. Song S, Li C, Li S, Cong X, Xue Y. Clinicopathological features and prognoses in younger and older patients with gastric cancer. *Onco Targets Ther*. 2017;10:4795-4802. doi:10.2147/ott.S144801

31. Wang Z, Xu J, Shi Z, Shen X, Luo T, Bi J, Nie M. Clinicopathologic characteristics and prognostic ofgastric cancer in young patients. *Scandinavianjournal ofgastroenterology*. Sep 2016;51(9):1043-9. doi:10. 1080/00365521.2016. 1180707

32. Song P, Wu L, Jiang B, Liu Z, Cao K, Guan W. Age-specific effects on the prognosis after surgery for gastric cancer: A SEER population-based analysis. *Oncotarget*. Jul 26 2016;7(30):48614-48624. doi:10. 18632/oncotarget.9548

33. Liu SS, Feng F, Xu GH, Liu Z, Tian YZ, Guo M, Lian X, Cai L, Fan DM, Zhang HW. Clinicopathological features and prognosis of gastric cancer in young patients. *BMC cancer*. Jul 2016;16478. doi:10. 1186/s12885-016-2489-5

34. Seo JY, Jin EH, Jo HJ, Yoon H, Shin CM, Park YS, Kim N, Jung HC, Lee DH. Clinicopathologic and molecular features associated with patient age in gastric cancer. *Worldjournal ofgastroenterology*. Jun 14 2015;21(22):6905-13. doi:10.3748/wjg.v21.i22.6905

35. Gurzu S, Kadar Z, Sugimura H, Bara T, Bara T, Jr., Halmaciu I, Jung I. Gastric cancer in young vs old Romanian patients: immunoprofile with emphasis on maspin and mena protein reactivity. *APMIS : actapathologica, microbiologica, et immunologica Scandinavica*. Mar 2015;123(3):223-33. doi:10. 1111/apm.12347

36. Schildberg CW, Abba M, Merkel S, Agaimy A, Dimmler A, Schlabrakowski A, Croner R, Leupold JH, Hohenberger W, Allgayer H. Gastric cancer patients less than 50 years ofage exhibit significant downregulation of E-cadherin and CDX2 compared to older reference populations. *Advances in medical sciences*. Mar 2014;59(1):142-6. doi:10. 1016/j.advms.2014.03.002

37. Karim S. Clinicopathological and p53 gene alteration comparison between young and older patients with gastric cancer. *Asian Pacificjournal ofcancerprevention : APJCP*. 2014;15(3):1375-9. doi:10 .7314/apjcp.2014. 15.3. 1375

38. Zheng C, Lu J, Huang C, Li P, Xie J, Wang J, Lin J. Clinicopathologic features and prognosis of gastric cancer in young patients. *Zhonghua wei chang wai ke za zhi [Chinesejournal ofgastrointestinal surgery]*. 2013;16(1):40-43. doi:10 .3760/cma.j.issn. 1671-0274.2013.01.011

39. Xu JB HY, Wu H, Zhang XH, Ma JP, Peng JJ, Chen CQ, Cai SR, Zhan WH. Clinicopathological features and prognosis ofyoung patients with gastric cancer. *Zhonghua wei chang wai ke za zhi*

[Chinesejournal ofgastrointestinal surgery] . 2013;16(1): 160-162 .
doi:10.3760/cma.j.issn.1671-0274.2013.02.018

40. Nguyen DK, Maggard-Gibbons M. Age, poverty, acculturation, and gastric cancer. *Surgery*. Sep 2013;154(3):444-52. doi:10. 1016/j.surg.2013.05.017

41. Liang YX, Deng JY, Guo HH, Ding XW, Wang XN, Wang BG, Zhang L, Liang H. Characteristics and prognosis of gastric cancer in patients aged ≥ 70 years. *Worldjournal ofgastroenterology*. Oct 21 2013;19(39):6568-78 . doi:10.3748/wjg.v19.i39.6568

42. Isobe T, Hashimoto K, Kizaki J, Miyagi M, Aoyagi K, Koufuji K, Shirouzu K. Characteristics and prognosis ofgastric cancer in young patients. *Oncology reports*. Jul 2013;30(1):43-49 .
doi:10.3892/or.2013.2467

43. Dhobi MA, Wani KA, Parray FQ, Wani RA, Wani ML, Peer GQ, Abdullah S, Wani IA, Wani MA, Shah MA, Thakur N. Gastric cancer in young patients. *Internationaljournal ofsurgical oncology*.
2013;2013:981654. doi:10. 1155/2013/981654

44. Qiu MZ, Wang ZQ, Zhang DS, Luo HY, Zhou ZW, Wang FH, Li YH, Jiang WQ, Xu RH.
Clinicopathological characteristics and prognostic analysis of gastric cancer in the young adult in China. *Tumour biology : thejournal ofthe International Societyfor Oncodevelopmental Biology andMedicine*. Jun 2011;32(3):509-14. doi:10. 1007/s13277-010-0145-2

45. Moelans CB, Milne AN, Morsink FH, Offerhaus GJ, van Diest PJ. Low frequency ofHER2 amplification and overexpression in early onset gastric cancer. *Cellular oncology (Dordrecht, Netherlands)*. Apr 2011;34(2):89-95. doi:10 . 1007/s13402-011-0021-0

46. Al-Refaie WB, Hu CY, Pisters PW, Chang GJ. Gastric adenocarcinoma in young patients: a population-based appraisal. *Annals ofsurgical oncology*. Oct 2011;18(10):2800-7 .
doi:10. 1245/s10434-011-1647-x

47. Geramizadeh B, Adeli OA, Rahsaz M, Mokhtari M, Sefidbakht S. Comparison ofthe Expression ofCell Adhesion Molecule Markers (E-Cadherin and Syndecan-1) between Young and Older Age Patients with Gastric Carcinoma. *Journal ofgastrointestinal cancer*. Sep 2010;41(3) : 193-6 .
doi:10. 1007/s12029-010-9149-9

48. Park JC, Lee YC, Kim JH, Kim YJ, Lee SK, Hyung WJ, Noh SH, Kim CB. Clinicopathological aspects and prognostic value with respect to age: an analysis of 3,362 consecutive gastric cancer patients. *Journal ofsurgical oncology*. Jun 1 2009;99(7):395-401 . doi:10 . 1002/jso.21281

49. Silva EM, Begnami MD, Fregnani JH, PelosofAG, Zitron C, Montagnini AL, Soares FA.
Cadherin-catenin adhesion system and mucin expression: a comparison between young and older patients with gastric carcinoma. *Gastric cancer : oficialjournal ofthe International Gastric Cancer Association and the Japanese Gastric CancerAssociation*. 2008;11(3): 149-59 .
doi:10. 1007/s10120-008-0468-5

50. Lai JF, Kim S, Li C, Oh SJ, Hyung WJ, Choi WH, Choi SH, Wang LB, Noh SH.
Clinicopathologic characteristics and prognosis for young gastric adenocarcinoma patients after curative resection. *Annals ofsurgical oncology*. May 2008;15(5) : 1464-9 .
doi:10. 1245/s10434-008-9809-1

51. Kim JH, Boo YJ, Park JM, Park SS, Kim SJ, Kim CS, Mok YJ. Incidence and long-term outcome ofyoung patients with gastric carcinoma according to sex: does hormonal status affect prognosis? *Archives ofsurgery (Chicago, Ill : 1960)* . Nov 2008;143(11):1062-7; discussion 1067.
doi:10. 1001/archsurg.143. 11. 1062

52. Sasao S, Hiyama T, Tanaka S, Yoshihara M, Yasui W, Chayama K. Clinicopathologic and genetic

characteristics of gastric cancer in young male and female patients. *Oncology reports*. Jul 2006;16(1):11-5.

53. Milne AN, Carvalho R, Morsink FM, Musler AR, de Leng WW, Ristimäki A, Offerhaus GJ. Early-onset gastric cancers have a different molecular expression profile than conventional gastric cancers. *Modern pathology : an official journal of the United States and Canadian Academy of Pathology, Inc.* Apr 2006;19(4):564-72. doi:10.1038/modpathol.3800563

54. Lim S, Lee HS, Kim HS, Kim YI, Kim WH. Alteration of E-cadherin-mediated adhesion protein is common, but microsatellite instability is uncommon in young age gastric cancers. *Histopathology*. Feb 2003;42(2):128-36. doi:10.1046/j.1365-2559.2003.01546.x

55. Kim DY, Ryu SY, Kim YJ, Kim SK. Clinicopathological characteristics of gastric carcinoma in young patients. *Langenbeck's archives of surgery*. Sep 2003;388(4):245-9.
doi:10.1007/s00423-003-0387-1

56. Theuer CP, Kurosaki T, Taylor TH, Anton-Culver H. Unique features of gastric carcinoma in the young: a population-based analysis. *Cancer*. Jul 1 1998;83(1):25-33.
doi:10.1002/(sici)1097-0142(19980701)83:1<25::aid-cncr4>3.0.co;2-d

57. Jee MS, Koo C, Kim MH, Choi C, Lee KM, Choi SK, Rew JS, Yoon CM. Microsatellite instability in Korean patients with gastric adenocarcinoma. *The Korean journal of internal medicine*. Jun 1997;12(2):144-54. doi:10.3904/kjim.1997.12.2.144

58. Maehara Y, Emi Y, Tomisaki S, Oshiro T, Kakeji Y, Ichiyoshi Y, Sugimachi K. Age-related characteristics of gastric carcinoma in young and elderly patients. *Cancer*. May 1 1996;77(9):1774-80. doi:10.1002/(sici)1097-0142(19960501)77:9<1774::Aid-cncr3>3.0.Co;2-c
